# Unidirectional Ion Sieve Enabling High-Flux and Reversible Zinc Anodes

**DOI:** 10.1021/acsnano.5c01103

**Published:** 2025-04-08

**Authors:** Zhiyuan Chen, Yifan Zhao, Ping Cui, Jiayan Zhu, Xuan Gao, Guanjie He, Xiaosu Yi

**Affiliations:** † Faculty of Science and Engineering, the University of Nottingham Ningbo China, Ningbo 315100, China; ‡ Department of Energy Storage Center, Shanghai Advanced Research Institute, Chinese Academy of Sciences, 99 Haike Road, Shanghai 201210, China; § State Key Laboratory of Superhard Materials, College of Physics, Jilin University, Changchun, Jilin 130012, PR China; ∥ Christopher Ingold Laboratory, Department of Chemistry, University College London, 20 Gordon Street, London WC1H 0AJ, U.K.; ⊥ Thom Building, Department of Engineering Science, University of Oxford, 17 Parks Road, Oxford OX1 3PJ, U.K.

**Keywords:** zinc-ion batteries, composites, zinc anode, aligned coating, biomimetic

## Abstract

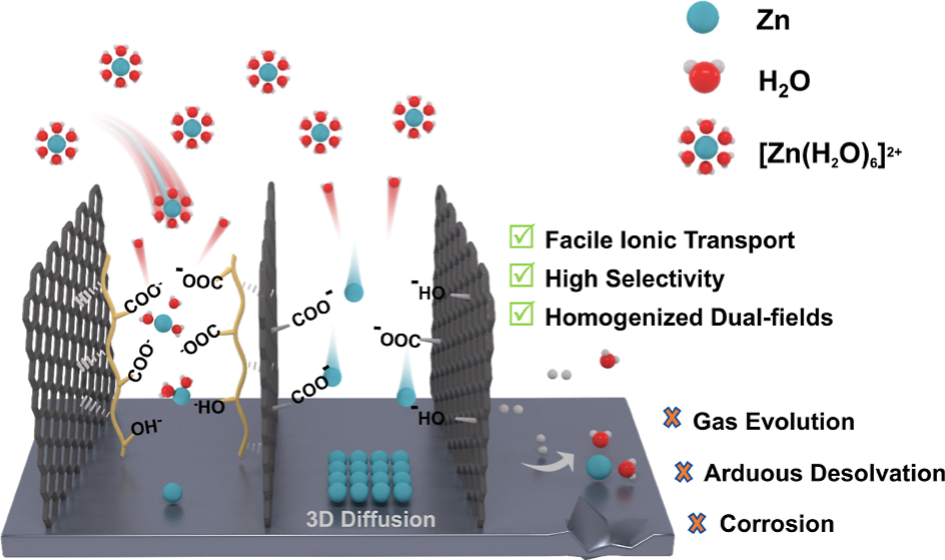

The longevity of aqueous batteries after scaling up is largely restricted by metal anodes (Zn, Al, and Mg). Parasitic reactions and uncontrolled dendrites dominate failure modes, especially at high current densities. To fully improve its reversibility, tailored surface chemistry and well-designed ion transport channels are simultaneously demanded. Here, inspired by the reticulated structure of the sea urchin shell, an aligned porous coating assembled from graphene oxide and sodium alginate is anchored on zinc anodes, termed a unidirectional ion sieve. As revealed by multiscale modeling and tests, this biomimetic layer produces a high surface area, creating low-tortuosity channels that greatly enhance transport kinetics and uniform distribution of ions. The introduction of an ion-conductive natural polymer enables a well-tuned hydration structure and ion selectivity, greatly alleviating aqueous side reactions. With the structural-functional integrity design, the decorated symmetrical cell presents reversible cycling for 1600 h, with a greatly reduced nucleation potential of 21 mV and high Coulombic efficiency. Aided by the Distribution of Relaxation Time tool, different electrochemical processes are deconvoluted to understand respective mechanisms, thereby providing a referable strategy for product scaling. In the end, a 7Ah Zn||VO_2_ pouch cell demonstrates stable cycling for over 500 cycles at 1 A·g^–1^, with the capacity retention over 90%.

## Introduction

Aqueous metal batteries represent one of the most encouraging electrochemical energy storage technologies in the postlithium era.^1^ Particularly in high-power applications, the decent ionic conductivity and inherent nonflammability of aqueous electrolyte promise manageable heat generation during fast cycling and provide genuine safety at large-scale installations. These distinctive features make it a competitive candidate over conventional lithium-ion batteries in grid-scale applications. Zinc metal has been extensively researched due to numerous merits, including a favorable electrode potential of −0.762 V, a competitive divalent capacity of 820 mA h·g^–1^, and its environmental friendliness and cost-effectiveness. However, the zinc anode suffers from a restricted operation lifespan even on a lab scale, not to mention the amplified challenges in product scaling. The most prominent failure modes result from dendrite penetration and parasitic gas evolution, both of which deteriorate as electrode sheet size increases.^2^ Particularly under high current densities, the unevenness of the zinc surface deviates electric field distribution,^3^ accelerating dendrite growth at tab edges and protrusions. Furthermore, the sluggish Zn^2+^ transport approaching the anode surface promotes preferential hydrogen evolution, which extensively affects the stability of interfacial reactions. The former leads to a progressive or abrupt short circuit upon cycling, while the latter undermines anode reversibility and causes fast decay due to electrolyte depletion. To develop a reversible zinc anode for robust and long-life aqueous batteries, surface engineering by protective coating is one of the cost-effective and scalable routes in the industry.^4^ The selection of a proper candidate typically includes the following criteria: (1) superior electrochemical stability and strong adhesion to the anode under aqueous environments; (2) high ionic conductivity, in this case zincophilicity to reduce mass transport barrier; (3) porous microstructure to enlarge surface area for facile nucleation and deposition; (4) ionic selectivity to prohibit adsorption of byproduct components; (5) withstand volume change upon plating and stripping. To fulfill these requirements, miscellaneous coating materials were adopted and gained exciting outcomes, including metal (oxide)-based,^5,6^ polymer-based,^7−9^ carbon-based,^10^ and others.^11−13^

Despite numerous improvements achieved, fulfilling all above-mentioned criteria simultaneously is not always straightforward. In this regard, biomimetic coating arises as a ready-to-fetch methodology, accumulated from years of natural selection and evolution. Researchers have already made attempts to apply these design protocols in the field of zinc anode protections.^14−16^ For instance, Huang et al. successfully anchored aligned bush-like coating on zinc by reduced graphene oxide.^17^ It shows ultralow voltage hysteresis upon cycling, as well as improved reaction kinetics thanks to high flux diffusion channels. Liu et al. used aramid nanofiber (ANF) and modified PVDF to fabricate composite coating (ANFZ), mimicking the composition of fibrous cartilage.^18^ The rigid ANF network provides high ion conductivity with decent mechanical strength. Coupled with the polymer phase, the electric field and Zn^2+^ concentration field are homogenized to alleviate dendrite growth. In similar cases, the use of building blocks with high surface area (such as nanofiber, MXene, or graphene) normally contributes to reduced plating overpotential and buffers volume change. However, the challenge remains. An increased surface area sensibly leads to impaired mechanical integrity of the coating, thereby constraining anode performances. This issue can be greatly renovated by introducing a functional polymer via strong cross-linking; however, the polymer phase would in turn elevate ion transfer resistance and polarization under high rates, sequentially causing sluggish Zn^2+^ plating kinetics. Therefore, to balance this unavoidable trade-off in biomimetic coating design for high-performance anodes, it undoubtedly requires exquisite consideration in terms of both structure design and interfacial chemistry.^19^

Herein, inspired by the intricate architecture of sea urchins, a species of echinoderms, a GO/sodium alginate (SA) composite coating (aGO-SA@Zn) is fabricated featuring a unidirectional porous structure. In this case, GO and SA are selected as building blocks because of the following reasons. GO readily undertakes self-assembly to construct 3D porous architectures in an aqueous environment^20^ and claimed to have low lattice mismatch^21^ with the zinc (002) plane besides graphene.^22^ SA, as a type of natural polymer, carries abundant oxygen-containing functional groups that form strong hydrogen bonds with the GO skeleton, enhancing its mechanical stability. Moreover, SA is reported to present high zinc ion transport efficiency compared with other polymers.^23^ Specifically, the COO- groups among G monomers of SA chains would cross-link with divalent zinc ions to form the “egg-box” aggregate, a model well reported in previous literature.^24^ The as-synthesized directional porous coating significantly reduces the tortuosity of the zinc diffusion path, facilitating better electrolyte infiltration and faster ion transport kinetics. Additionally, the water-repellent yet zincophilic coating enhances desolvation of Zn^2+^ via electrostatic force, decreasing nucleation barrier and mitigating water-associated side reactions. The synergistic effects of structural alignment and interfacial ion selectivity prompt homogeneous Zn deposition under high current conditions, effectively suppressing side reactions and enhancing cycling longevity. In this way, the coating is termed as “unidirectional ion sieves”. The corresponding symmetrical cell demonstrates remarkable cycling stability for over 1600 h at 1 mA h·cm^–2^, along with markedly reduced polarization during rate tests. A 7Ah pouch cell assembled with aGO-SA@Zn||VO_2_ exhibits an impressive anode reversibility and a capacity retention (92.7%) for 500 cycles under 3C discharge conditions. This work integrates macroscopic biomimetic architecture design with tailored interfacial chemistry at the microscale, proposing a valuable scaling-up concept for advanced zinc-ion batteries.

## Results and Discussion

### Morphology and Characterization

**Figure 1:**
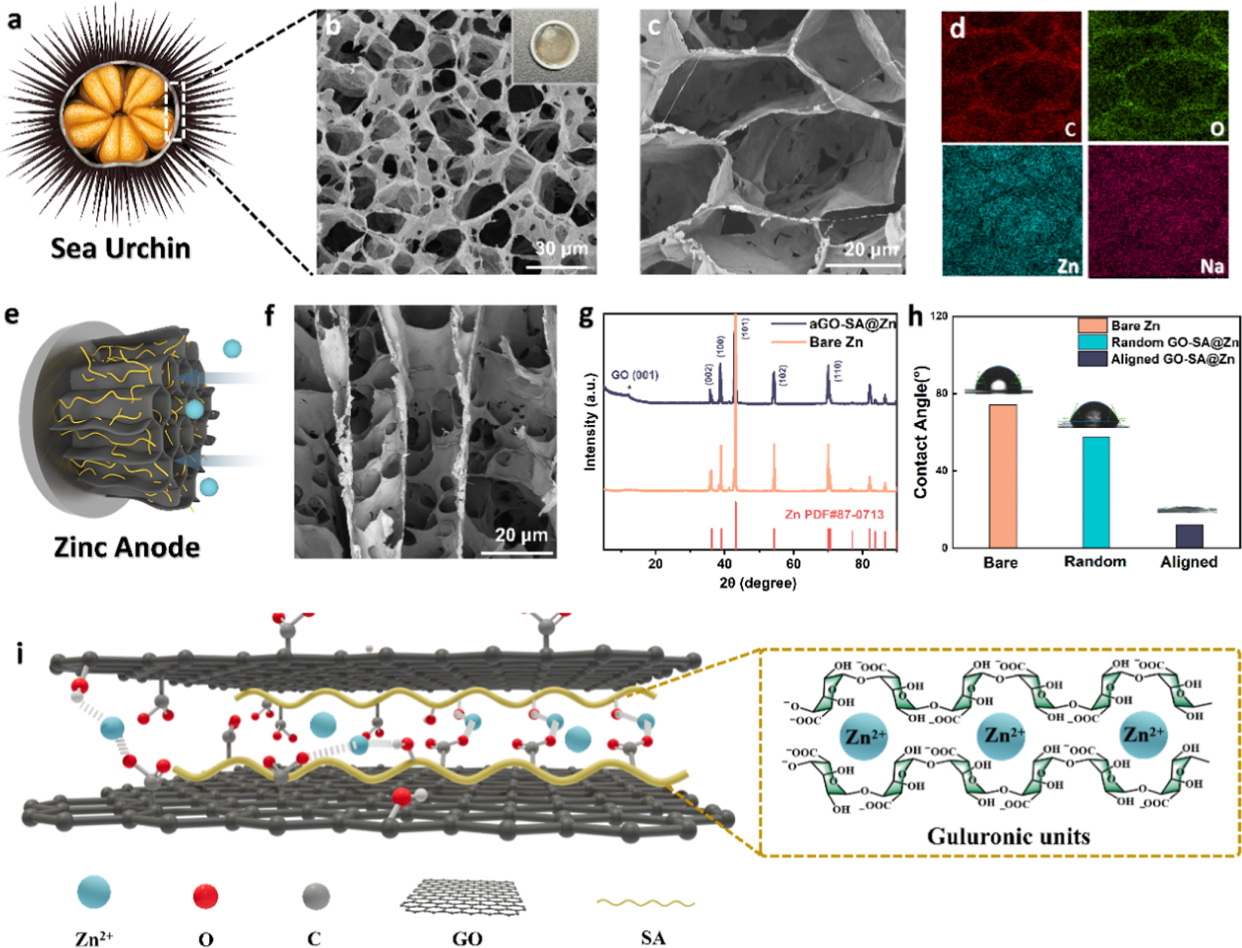
(a) Cross-sectional anatomy of a sea urchin, with its shell structure in a rectangle (Credits to graphicsrf on Vecteezy under a free Creative Commons Attribution License).^25^ (b) Top-view scanning electron microscope (SEM) images of the biomimetic reticular structure of the aligned GO-SA@Zn. The inserted image shows the sample in the coin cell holder. (c) Top-view SEM image with higher magnification. (d) EDS image with respect to the top view image. (e) Illustration diagram of the aligned GO-SA-coated zinc anode, with facile zinc ions infiltration. (f) Side-view SEM images of the sample. (g) XRD pattern of the as-synthesized GO-SA composite, pristine zinc, and zinc standard pattern. (h) CA of bare zinc, random GO-SA-coated zinc, and aGO-SA-coated zinc surface in a 2 M ZnSO_4_ electrolyte (from left to right). (i) Schematic diagram of intramolecular forces between lamellae GO and SA, where the magnified image depicts the cross-linking of the G monomer with divalent Zn^2+^.

Figure 1a depicts the cross-sectional anatomy of a sea urchin,^25^ with a calcite skeleton that builds up typical reticular architecture. The oriented pores have diameters ranging from a few micrometers to several hundred micrometers, which play an essential role in accelerating gas and nutrient exchange with aqueous surroundings.^26^ For this reason, efficient respiration and metabolism are achieved. Inspired by its subtle structure, GO and SA building blocks are assembled as a biomimetic aligned GO-SA coating (Figure 1b), denoted as aGO-SA, which presents a light brown color compared to the dark brown of pure GO. The schematic diagram of the preparation method for aGO-SA is given in Figure S1. In a nutshell, the interpenetrated molecules are repelled by the growing ice crystal cylinders during the directional-freezing process, arranging along a perpendicular direction and restacking to form cellular networks. Figure 1c with a magnified view shows that the pores within the skeleton are in the range of 20–30 μm, serving as fast transport channels for homogenized ion flux. Figure S2 magnifies the joint part of the GO sheets, demonstrating decent structural continuity and elastic strength. These features are reported to create a highly effective surface area and buffer volume expansion during the plating.^27^ Energy dispersive X-ray spectroscopy (EDS) is performed with respect to the domain in Figure 1c. The distinct element contour suggests an abundance of oxygen-containing functional groups covering the GO skeleton. Through intramolecular hydrogen bonding, GO nanosheets and SA chains would establish robust interconnected hollow networks (Figure 1e), serving as a stable ion delivery interphase that allows the fast absorption and transfer of divalent metal ions. The intramolecular interactions in detail are proposed in Figures 1i and S3, which show various bonding types, including hydrogen bonds between GO and SA chains, the “egg-box” gelation structure in between G monomers of SA and divalent Zn^2+^, as well as coordination of GO and Zn^2+^ mainly by COO^–^ groups.^24^ Additionally, the uniform dispersion of zinc and sodium elements within porous channels suggests an even distribution of SA molecules. This ensures extensive interactions between the active species and the coating. Figure 1f verifies the predominant upward distribution of graphene sheets. These anisotropic sheets compose the bush-like region, creating an interim boundary between the electrolyte and zinc anodes, boosting electrolyte infiltration, and increasing the effective surface area at high current density. Figure S4a narrates that the aligned structure can sustain uniformity exceeding 100 μm thickness. Figure S4b clearly shows the multilayer GO, with a lateral size of approximately 20 μm. It also highlights the zincophilic nature of the hybrid film at the interface, showing a smooth and tight connection at the junction. It is proposed that mildly acidic GO from the modified Hummer’s method would be spontaneously reduced by zinc and undergo self-assembly.^28,29^ Consequently, GO layers are tightly bonded to the zinc metal surface, resulting in a well-defined, smooth margin. The introduction of SA not only offers ample oxygen-containing polar groups at the coating surface but also serves as a gelation agent and binder in case the pure GO skeleton may easily shrink and collapse after freeze-drying.^30^ It also enhances the antidissolution stability of GO sheets in an aqueous environment, as underpinned by the soaking experiment in Figure S5 that GO coating without this natural polymer would easily peel off from the zinc surface. In an opposite way, GO sheets enable the construction of reticulated channels for fast ion transfer, simultaneously preventing the degradation of SA gel^24^ and buffering the volume expansion, as reported.^31^ Moreover, the highly porous skeleton offers a number of electrochemically active surfaces, contributing to reduced activation barriers and faster reaction kinetics. In conclusion, the interplay of inorganic GO skeleton and organic SA chain contributes to comprehensive electrolyte–electrode interactions and robustness of the coating, which hopefully ensures high-rate capability and stability of the electrode. The XRD graph shown in Figure 1g exhibits the characteristic peak of the GO (001) plane at around 11°compared with pristine zinc, manifesting its intrinsic polar functional groups. It is also noteworthy that the (101) crystal plane dominates the bare zinc surface, along which plane dendrites tend to form. As a major indicator of electrolyte infiltration kinetics, contact angle (CA) is first measured in 2 M ZnSO_4_ to assess surface wettability. According to Figure 1h, aligned GO-SA-coated zinc shows a significantly reduced CA of 11.9° compared to 74.2° for bare zinc. The improved compatibility of the coated sample gives credit to the following two aspects. First, both SA and GO have polar functional groups such as –COO, which counteracts the hydrophobic effect of the sp_2_ carbon ring insensitively. Second, the directional microstructure in aligned coating promotes the absorption of aqueous electrolytes thanks to capillarity force,^17,31^ benefiting the permeation of zinc ions into the electrode and homogenizing the zinc ions influx simultaneously. This assumption also clarifies why random groups show an increased CA of 57.6°, despite identical coating composition; the horizontal sheet arrangement establishes tortuous channels and hinders ionic diffusion. The improved wettability, as correlated by classic nucleation theory,^32^ is also beneficial to reducing the heterogeneous nucleation Gibbs free energy, thereby facilitating reaction kinetics. Similar findings are also obtained in other literature to verify these structural advantages.^17,33^

### Fast Ionic Transport and Reaction Kinetics

Aqueous zinc-ion battery is born for high-rate applications, and thus, an artificial solid electrolyte interphase (SEI) layer is coated to protect the zinc surface from devastating protrusions and parasitic reactions. However, the introduction of a protective layer brings about an extra mass transfer boundary, susceptible to deteriorating concentration polarization upon fast charging.^34^ In the context of aligned GO-SA coating, the ionic transport behaviors across electrolyte-anode multiphases are investigated via both first-principles calculations and electrochemical analytical measures.

**Figure 2:**
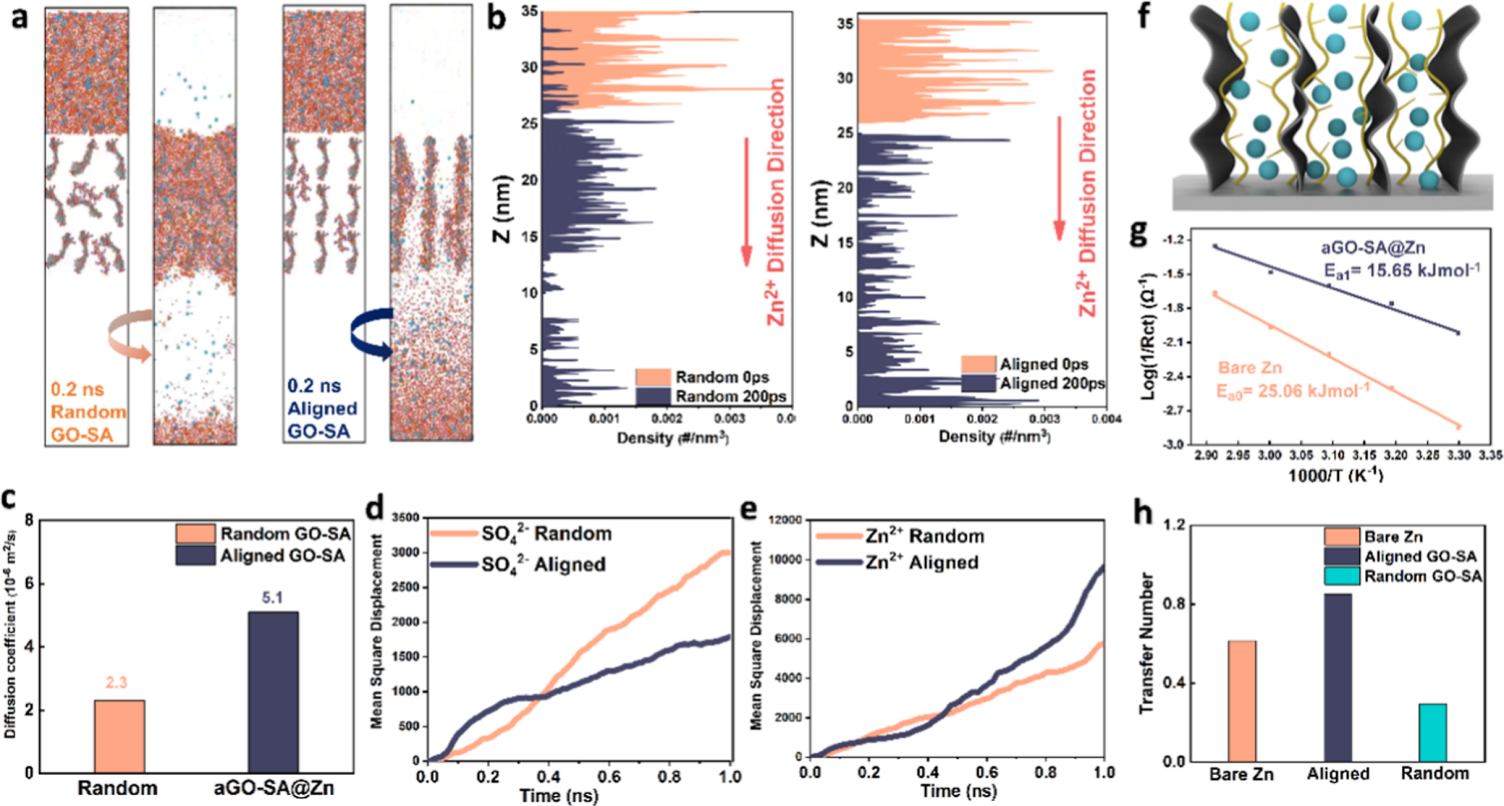
(a) Snapshot of the numerical results of zinc flux through (left) random GO-SA coating and (right) biomimetic GO-SA ion sieves before and after 0.2 ns; (b) the corresponding atom density distribution along the zinc flux direction. (c) Calculated diffusion coefficient by the MD simulation result. (d) MSD result of the sulfate ion within a time interval of 1 ns; (e) MSD value of zinc ions within 1 ns. (f) Demonstration model of the as-fabricated oriented GO-SA composite and its possible interaction pattern. Dark refers to GO sheets, blue refers to zinc ions, and brown refers to SA polymers. (g) Activation energy extrapolation from Arrhenius law for bare zinc in orange and aGO-SA-coated zinc in dark blue. (h) Zn^2+^transference number of bare zinc, random GO-SA, and aligned GO-SA-coated zinc.

To justify the structural superiority of sheet alignment, molecular dynamics (MD) is applied to visualize the corresponding molecular flux in comparison to random GO-SA coating. The simulation box contains 17,247 atoms in total. It can be observed from Figure 2a that, in a duration of 0.2 ns, electrolyte molecules migrate considerably slower in the random group than in the oriented group shown in the right-hand side. The random group exhibits an ion-gathering effect in between sheets as expected, with a limited number of molecules reaching the bottom electrode surface. The animated result in the Supporting Information Video S1 provides a more comprehensive view. For further interpretation, Figure 2b quantitatively tabulates ion density distribution across migration routes. The orange lines stand for the original distribution and the blue ones for the condition after 0.2 ns. Molecule density within the random coating region makes up the greatest proportion of all, while for the oriented group, the density peak shifts downward and reaches maxima near the electrode surface. Moreover, it is estimated that the diffusion coefficient for the aGO-SA coating reaches 5.1 × 10^–6^ m^2^/s, which increases by 121% compared with the random group. The improvement in mass transfer would remarkably decrease concentration polarization to improve overall energy efficiency.^35^ Under fast charging–discharging, aligned GO-SA also ensures fast passage and uniform distribution of reactive species so as to avoid ion gathering and possible parasitic reactions. Ultimately, the enhanced reaction kinetics would slow electrode degradation, resulting in a longer cycle life. The mean square displacement (MSD) for both sulfate ion and zinc ion is also calculated. Over a 1 ns time frame, it is observed that Zn^2+^ transport behavior in two samples does not greatly differ at the initial stage, mainly because of burdensome solvated aggregates. Thereafter, zinc flux through aligned interphase apparently exceeds that of random coating, and both groups present greater MSD values than their sulfate ion counterpart because of interfacial sieving. In Figure 2d, nevertheless, the sulfate ion shows an interesting intersection at around 0.4 ns. Prior to this point, sulfate ions in the aligned sample demonstrate a greater average square distance (MSD), likely due to the capillarity of the highly porous structure discussed earlier. After 0.4 ns, however, the diffusion rate appears to slow down as indicated by the flattened slope, essentially caused by electrostatic repulsion from oxygen-containing functional groups in both GO and SA. Before the sulfate ion further reaches the Helmholtz layer to produce inert byproducts,^36^ its mobility can be effectively suppressed. Therefore, the hybrid coating can not only accelerate ionic transport but also act as an ion sieve that prohibits the passage of unwanted species.

In terms of zinc activation kinetics, the energetically demanding desolvation of hexahydrated zinc ions is generally recognized as the rate-limiting step.^37^ Activation energy (*E*_a_) is thus calculated (Figure 2g) based on Arrhenius’ law, deriving from temperature-dependent impedance values from the Electrochemical Impedance Spectroscopy (EIS) technique. It can be seen that the activation energy for aGO-SA (15.65 kJ·mol^–1^) is 37.5% lower than that of bare zinc (25.06 kJ·mol^–1^). It implies that the aligned GO-SA composite significantly accelerates zinc ion desolvation process approaching the electrode surface, which contributes to facile ionic transport in the bulk interface. Apart from boosting the desolvation process, the zincophilic layer would naturally repel negatively charged molecules (H_2_O and SO_4_^2–^) away from the bulk surface, thereby synergistically reducing Zn ion diffusion resistance by enhancing selectivity. A good illustration comes from the cation transference number (*t*_*+*_), which primarily represents the conduction efficiency of Zn ions across the electrolyte-coating interface (Figure 2h). For pristine zinc, the transfer number is 0.61; while for aGO-SA-coated zinc, it increases to an impressive 0.85 by 40%, which means that Zn^2+^ plays the major role in liquid phase current flow.^38^ This enhancement is primarily owing to the larger specific surface area provided by the 3D-oriented coating, which hopefully minimizes the concentration difference and promotes high power performance. By contrast, this value for random coating drops to 0.29. The sluggish ion flux is directly ascribed to the long and tortuous pathways that block Zn ion mobility.^14^ Aside from longer transport channels, the random arrangement of 2D sheets also alters the electric field distribution that may indirectly impede the movement of Zn^2+^ ions toward the plating surface. As revealed by the simulation result in Figure S27, the random graphene layers trigger the “tip effect” at the graphene edges, which creates the electrically congregated region amidst horizontal sheets. This intense area tends to trap Zn^2+^ ions and retard their movement along the path, making it hardly approach the electrode surface. Consequently, the concentration field modeling shows great variation after a characteristic time of 1s. This sensibly leads to greater concentration polarization and lower reaction kinetics predominantly in high-rate conditions.

Figure 2f shows a simplified model of the aGO-SA coating: GO sheets establish fast electric and ionic conduction framework, and entangled SA molecules provide connective strength through hydrogen bonds. In a synergistic manner, both SA and GO tend to attract divalent Zn ions due to the presence of a carboxylic group.^39^ Alternatively, these oxygen-containing groups would repel electronegative molecules during zinc diffusion and desolvation, which is believed to greatly alleviate water-induced corrosion and gas evolution during deposition. Accordingly, this ionic selectivity feature would effectively promote reversibility and prolong the cycle life of zinc anodes. To summarize, dual functionality in terms of rapid transport kinetics and ionic selectivity is effectively achieved with this biomimetic design of coating, which essentially results from the synergistic effects of structural orientation and surface chemistry.

### Interfacial Chemistry and Deposition Behaviors

**Figure 3:**
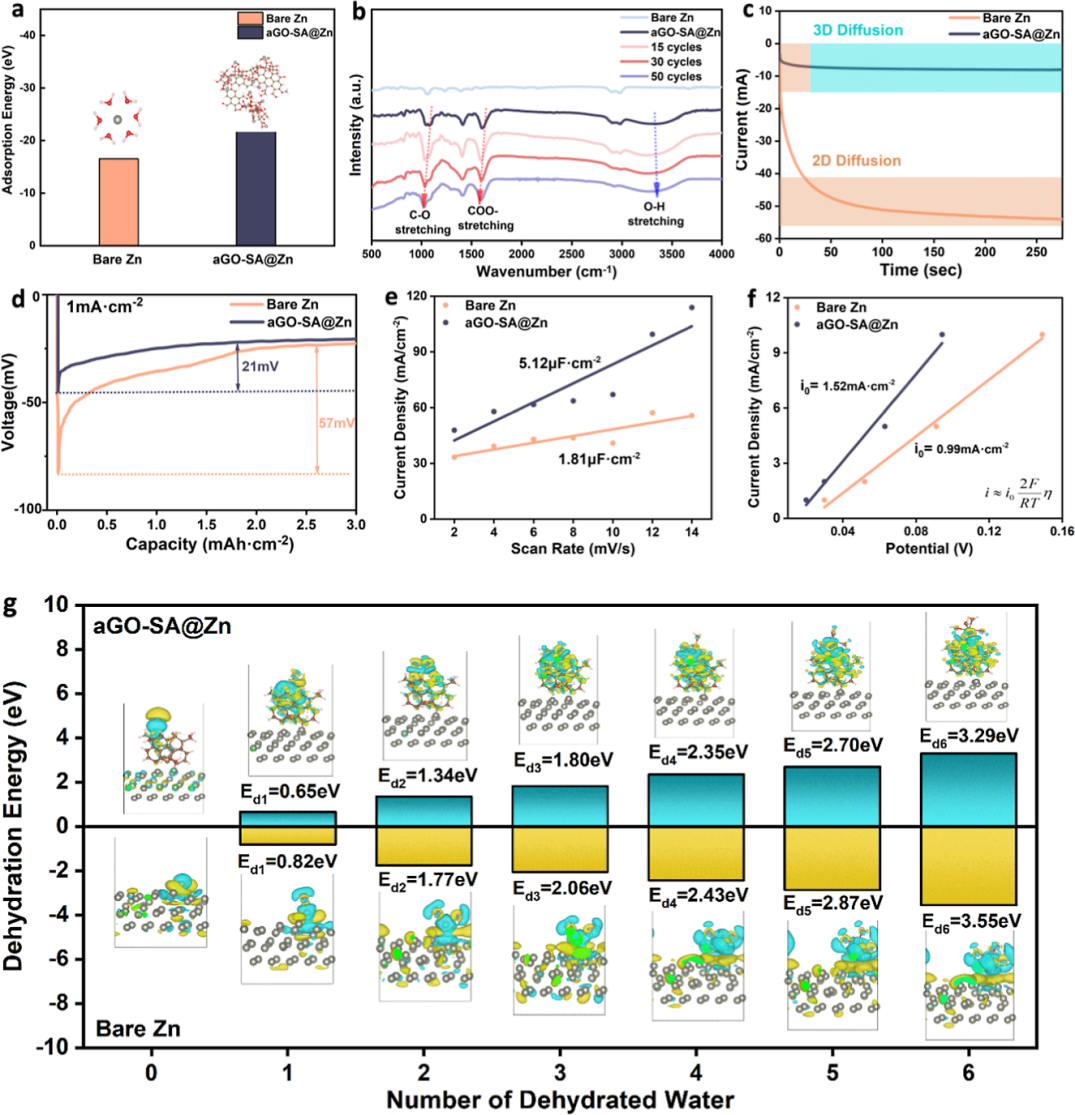
(a) DFT calculation of adsorption energy of Zn–H_2_O and Zn-GO/SA with respective modeling structures. (b) Ex-situ FTIR diagrams of pristine/coated zinc after different cycles, as well as corresponding peak shifts. Cycling condition is 1 mA·cm^–2^, 1 mA h·cm^–2^; (c) Chronoamperogram of bare Zn and aligned GO-SA-coated zinc electrodes at 150 mV overpotential for 300 s. Different colors represent 2D/3D diffusion approaching the zinc surface. (d) Nucleation overpotential upon plating on different copper electrodes at 1 mA·cm^–2^. (e) EDL capacitance interpolation of the Zn symmetrical cell with or without coating. (f) Exchange current interpolation of exchange current density from the modified Butler–Volmer equation. (g) Stepwise dehydration energy of [Zn (H_2_O)_*n*_]^2+^, respectively, at aGO-SA@Zn and bare zinc surface. Inserted images are differential charge density diagrams at different solvated states.

Beyond structural factors, the interfacial property of the functional coating also significantly affects the reaction pathway and plating kinetics of zinc deposition. DFT calculation is first performed with respect to the energy-intensive dehydration process (Figure 3a). The binding energy of Zn^2+^ to aGO-SA (−22.7 eV) is evidently lower than that of water molecules (−16.5 eV). Upon entering the catalytically active layer, the negatively charged GO-SA surface would readily adjust the electrostatic environment surrounding Zn^2+^ and modify the hexahydrated coordination complex.^23^ The stepwise dehydration energy ([Zn (H_2_O)_*n*_]^2+^, where *n* is an integer from 0 to 6) is also simulated for both samples. Particularly, the dehydration energy for removal of 6 water molecules is 3.29 eV for aGO-SA@Zn, which is apparently lower than 3.55 eV of the bare zinc surface. Consequently, the arduous desolvation process can occur in a more kinetically favorable pathway before reaching the inner Helmholtz plane (IHP), giving rise to lower activation energy and enhanced electroplating kinetics. The functional groups of the hybrid coating are also characterized with ex-situ FTIR (Figure 3b). The typical peaks at around 3230, 1605, and 1045 cm^–1^ are assigned to alcoholic O–H stretching, COO– stretching, and C–O stretching, respectively. With consecutive 50 cycles at 5 mA·cm^–2^ current density, the O–H stretching vibration peak blue shifts to 3267 cm^–1^, while COO– exhibits red shifts to 1590 cm^–1^, ascribed to coordination with Zn^2+^ with the aforementioned egg-box structure.^24^ In order to unveil the composition distribution and molecular interaction within the coating, high-resolution X-ray photoelectron spectroscopy (XPS) is performed. The atomic content of oxygen is 38.7%, while carbon takes up 42.7%, confirming its highly electronegative and hydrophilic surface nature. From Figure S9, the O 1s spectra of the pristine GO-SA sample can be split into three distinct bonding states, which are O–H (535.58 eV), C=O (532.88 eV), and C–O (531.75 eV). These characteristic polar groups are mutually shared by GO and SA, allowing the bridging of the two constituents via hydrogen bonds and collective absorption for zinc ions (Figure S3). After cycling for 150 cycles, the peak binding energy shifts to a lower value. The presence of a sharp O–Zn peak at 530.62 eV reveals a change in localized electron distribution owing to coordination with Zn^2+^, which shields the effective attraction felt by the O 1s electron from oxygen nucleus. Additional proof is shown in Figure S10; the Zn 2p_1/2_ binding energy of the coated sample is 1022.87 eV compared with 1020.89 eV of bare zinc, indicative of reduced electron cloud density due to bonding with functional groups. These spectra collectively substantiate the formation of a GO/SA-Zn^2+^ coordination aggregate, serving as stable intermediates in the catalyzed dehydration process. As a result, zinc ions would more facilely reach the electrode surface for the subsequent charge transfer step, reducing the activation energy barrier and enhancing the overall efficiency.

Chronoamperometry (CA) is also conducted on Zn||Zn symmetric cells to investigate surface diffusion and nucleation behaviors. There are mainly two types of diffusion modes. The 2D pattern refers to diffusion of species that is confined to lateral dimension, while the 3D pattern occurs where species diffuse freely in three dimensions. They are distinguishable by the Cottrell equation.^40^ In the 2D region, the current density bears a linear relation with *t*^–1^, and for 3D diffusion, the current decay is proportional to *t*^–1/2^. With a step potential of −150 mV, the coated zinc surface exhibits a responsive current of 6 mA. After transitory 2D diffusion of approximately 11 s, it quickly converts to steady-state 3D diffusion with a current of 8 mA, as linear fitting illustrates in Figure S12. The 3D behavior indicates direct reduction of Zn^2+^ to solid phase approaching the electrode surface, which is sensible in a porous electrode with a high surface area and plentiful active sites, according to previous literature. For 2D diffusion, which occurs mostly in the bare zinc group shown in Figure 3c, the current continuously increases in a duration of 500s. The adsorbed Zn^2+^ tends to migrate and aggregate at the most energetically favorable spots, typically at the primitive nucleus or initial deposition layer, as a way to reduce surface energy.^41^ Subsequently, the deposits tend to form a loose structure and induce localized dendrite growth. Nucleation overpotential is tested on the Zn||Cu asymmetrical cell shown in Figure 3d. Aligned GO-SA-coated zinc exhibits a lower overpotential of 21 mV compared with 57 mV for bare zinc. The lower nucleation overpotential implies that less additional voltage is required to overcome thermodynamic barriers for stable nucleus formation.^42^ The immediate benefit would be lower activation polarization and higher energy efficiency, which align with *E*_a_ values, as previously calculated. Regarding the decreased nucleation barrier, three reasons may be taken into account. First, desolvation through coating is achieved stepwise instead of taking place altogether at the outer Helmholtz plane; therefore, more desolvated Zn^2+^ would approach the IHP for direct charge transfer, thus becoming less energy demanding. Second, the oriented carbon skeleton creates a high surface area with more deposition sites available to Zn^2+^, while a fast transport channel ensures high and uniform ionic flux, maintaining a constant electric double layer (EDL).^43^ These prerequisites increase the likelihood of nucleation events and stable nucleus formation. Third, the presence of an inherent oxide layer in IHP can bring about an extra nucleation barrier to bare zinc. According to classic nucleation theory, the nucleation overpotential inversely affects the critical nucleus size (r*) by influencing the Gibbs free energy of nucleation^44^
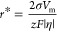
where σ is the surface energy, *V*_m_ is the molar volume of the deposited metal, *z* refers to charge number, *F* stands for Faraday constant, and η is nucleation overpotential. In the scenario of GO-SA-coated zinc, the lower overpotential encourages the formation of fewer and larger nucleus.^45^ As growth prioritizes the formation of nucleation sites, activated zinc tends to deposit on the existing nucleus in a more controllable manner. For this reason, stable plating and higher Coulombic efficiency (CE) can be accomplished.^42,46^ Moreover, the lower nucleation overpotential tends to minimize the risk of competitive hydrogen evolution,^47^ promoting a stable dual-field environment and effectively extending cycle life. To further validate the enlarged deposition area, non-Faradaic EDL capacitance (EDLC) is measured by fitting cyclic voltammetry (CV) curves at different scan rates, as shown in Figures 3e and S11. The aligned group has a capacitance of 5.12 μF·cm^–2^, which is higher than 1.81 μF·cm^–2^ of bare zinc. According to the definition equation *C* = ε*A*/*d*, the capacitance is directly proportional to the effective surface area of the electrode.^48^ In the case of bare zinc, EDLC is established by water and other specifically adsorbed molecules at the near surface, and it typically possesses a thickness of around 2–5 Å.^43^ The diffusive layer spreads out further into the bulk electrolyte. For aligned GO-SA coating, however, the IHP may redistribute and extend into the coating surface, attracting Zn^2+^ to pack more densely. The corresponding diffusive layer would also be compressed to reduce the potential drop across the interface.^49^ These shifts in double-layer structure, along with abundant active species in aligned coating, significantly reduce the nucleation barrier and promote uniform zinc deposition.^50^ Exchange current density is also interpolated by the modified Butler–Volmer equation (Figure 3f). The aligned coating sample shows higher exchange current density (1.52 mA·cm^–2^) than bare zinc (0.99 mA·cm^–2^), which implies faster electrochemical reaction kinetics at equilibrium.^51^ This improvement would also validate the higher activity of coating with an enlarged surface area.

**Figure 4:**
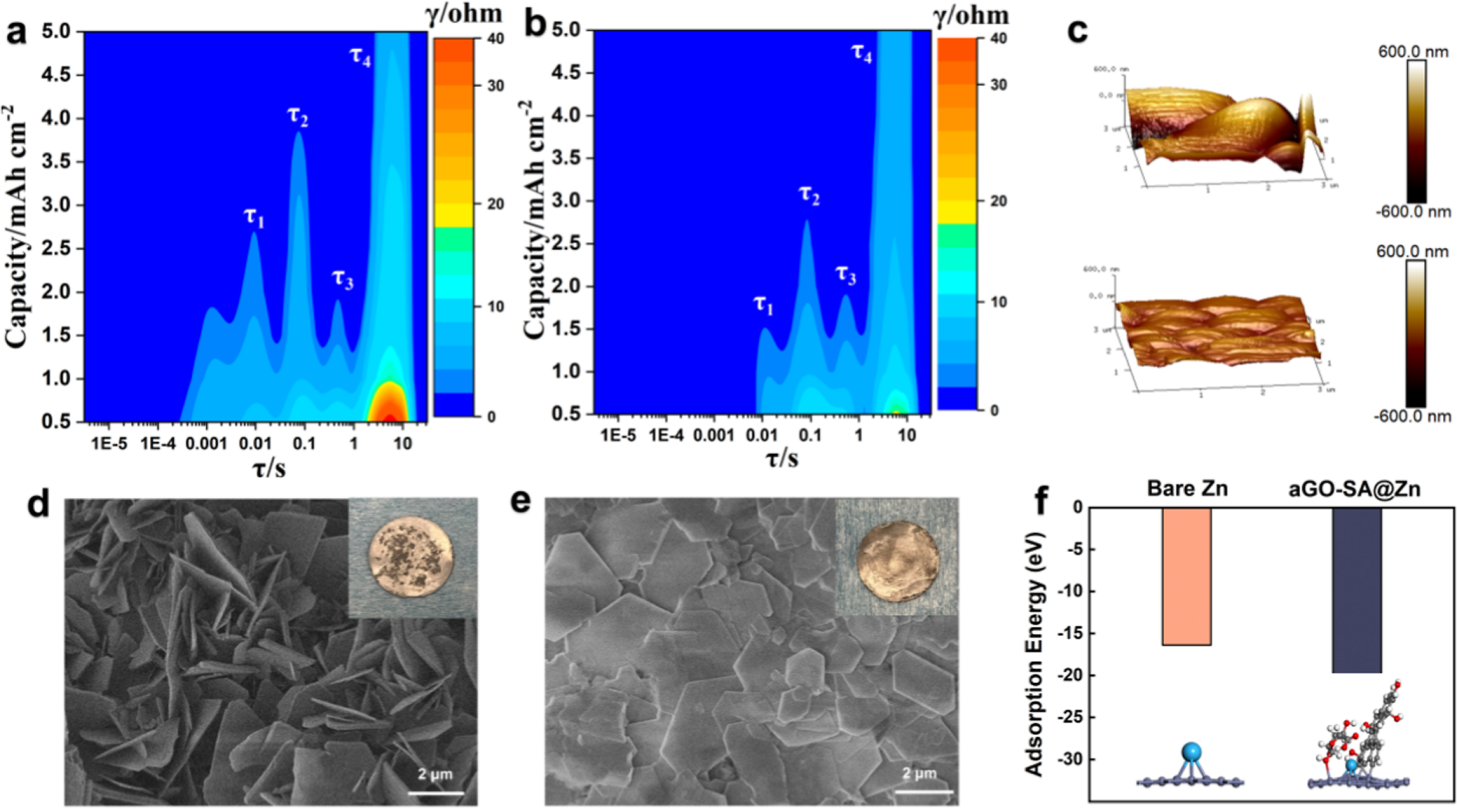
DRT analysis contour mapping of (a) bare zinc sample and (b) aligned GO-SA sample with continuous deposition from 0.5 to 5 mA h·cm^–2^. Annotations refer to different relaxation time constant. (c) AFM images of top: bare zinc bottom: aligned GO-SA zinc (coating removed) after 10 cycles at 0.5 mA·cm^–2^, 0.5 mA h·cm^–2^. Top SEM views of (d) bare zinc and (e) aligned GO-SA zinc (coating removed) after 10 cycles at 0.5 mA·cm^–2^, 0.5 mA h·cm^–2^. (f) Adsorption energy of Zn–Zn(002) and Zn-GO/SA@Zn(002), respectively, from DFT calculation, coupled with the schematic model.

Distribution of relaxation times (DRT) analysis from EIS data is performed to scrutinize individual electrochemical processes without relying on the predefined electric circuit model.^52^ By mapping the resistance evolution of bare zinc and coated zinc under a continuous deposition of 5 mA h·cm^–2^, interfacial properties and deposition kinetics can be closely traced.^53^ As shown in Figure 4a,b, four distinct relaxation time constants can be identified at around 0.01, 0.1, 1, and 10s, respectively, denoted as τ_1_ to τ_4_. These explicit time domains represent specific electrochemical processes: absorption and desolvation of Zn^2+^ (*R*_ads_), surface migration and solid-state diffusion of zinc-associated intermediates during zinc electrocrystallization (*R*_ssd_),^54^ charge transfer across the interface boundary (*R*_ct_), and diffusion within the porous electrode (*R*_diff_).^55,56^ Overall, it is evident that impedance values for both samples decrease as deposition proceeds, which can be attributed to factors such as enlarged electrode surface with more active sites, enhance electrode–electrolyte wettability, and increased local temperature.^57^ Specifically, the improvement in R_ads_ at τ_1_ reflects accelerated desolvation, promoted by the negatively charged polar groups of SA, which is already elaborated in the preceding part. For solid-state diffusion (τ_2_), bare zinc shows more prominent 2D diffusion and thus a comparatively higher impedance value, which is consistent with chronoamperometry results. The similar charge transfer (*R*_ct_) values at τ_3_ for both samples suggest that GO-SA coating does not noticeably enhance interfacial electron transfer, as the GO sheet with abundant polar groups greatly weakens its conduction of delocalized π bond electrons. The most notable improvement of aligned GO-SA coating lies in the substantial reduction of diffusion impedance (τ_4_), as seen in the deep red region in Figure 4a. The initial *R*_diff_ for the coated sample at 0.5 mA h·cm^–2^ is about 20 Ω, much lower than the bare zinc of 45 Ω. Additionally, the coated zinc reaches an equilibrium state after around 2.5 mA h·cm^–2^ deposition, whereas the impedance of bare zinc continues to change even after 5 mA h·cm^–2^. There are two main contributors. First, GO-SA coating with directional pores enhances electrolyte infiltration kinetics and selectively facilitates Zn^2+^ transport over solvent molecules. With high ionic flux and sieving effect, the ionic conduction pathway sensibly establishes quickly. Second, the increased surface area results in a thinner and more compact diffusive layer, as discussed previously. This shorter diffusion distance and diminished concentration gradient would mitigate mass transfer resistance. Moreover, a peak at around 10^–3^ seconds, highlighted in Figure S13, is observed for bare zinc. This relaxation domain is typically associated with SEI and can be rationally ascribed to the zinc oxide layer.^58^ Analogous to SEI, this layer at the IHP inevitably causes a potential drop that contributes to a higher nucleation barrier, as further evidence for increased overpotential.

The deposition morphology with a capacity of 0.5 mA h·cm^–2^ is examined using SEM. As shown in Figure 4d, the bare zinc sample exhibits loose plating and rampant dendrite growth in the vertical direction. In contrast, the aligned GO-SA sample displays flat and dense zinc flakes with an average size of 2 μm after removal of the coating, which implies induced deposition in preferential orientation (Figure 4e). From the inserted capture, it can be seen that a thin layer of carbon clings to the zinc surface arising from tight binding between them. The top view image of the coating after cycling (Figure S14) shows that it maintains a unidirectional structure, leaving the pores unblocked. Notably, zinc deposition occurs not only at the anode but also partially on the surface of hybrid skeletons. This phenomenon is dissimilar to other literature that uses reduced GO as the functional layer,^17^ where no apparent zinc deposits are observed on the coating. The difference can be explained by the increased number of polar groups on GO-SA surfaces compared with reduced GO, which boosts desolvation and promotes facile zinc deposition at edges, defects, and high curvature^59^ active sites. AFM analysis is applied to show a difference in surface roughness (Figure 4c). Same as SEM results, the coated sample presents a flattened deposition layer, with an average roughness of 33.9 nm compared with 297 nm of bare zinc. The surface area of the coated sample also markedly reduces to 10.1 μm^2^ in contrast to 14.7 μm^2^ of bare zinc, indicating uniform and compact deposition. This reduction in the electrode surface area also supports EDLC and DRT results, indicating that the lowered charge transfer resistance is primarily due to sufficient active sites provided by aligned GO walls rather than volumetric expansion of zinc during cycling. Ex situ XRD results track the evolution of the zinc surface texture (Figure S15). It shows that after continuous 70 cycles, growth along the zinc (002) crystal plane is greatly favored, as evidenced by the strengthening peak at 37°. Specifically, the primitive *I*_(002)_/*I*_(101)_ ratio is around 0.058 and increased 10-fold to 0.575 after 70 cycles. This observation conforms to SEM morphology results that aligned GO-SA coating stimulates uniform and dense zinc deposition along the (002) plane.^60^ DFT result justifies this facilitated effect as modeled and calculated in Figures 4f and S16. Compared to bare zinc (002), Zn^2+^ indeed shows greater affinity (20.2 eV) toward aligned GO-SA-coated zinc (002), indicating the preferential (002) epitaxial growth direction induced by the aligned coating. In this case, fewer dendrites and a longer cycling span can be realized.

### Improved Reversibility

From the material’s perspective, dendrite growth and parasitic reactions are the two predominant challenges impeding long-term cycling of zinc aqueous batteries. Dendrite growth cumulatively leads to a short circuit, while hydrogen evolution disrupts the electrode–electrolyte interface and distorts localized electric field distribution.^61^ Moreover, byproduct formation, primarily basic zinc sulfate, is accelerated either due to a change of regional ion species (OH^–^) or intensified local reaction kinetics. In the short term, surface byproducts would appear as transfer resistance to compromise CE, while over time, electrolyte depletion and subsequent clustered dendrite growth would cause sudden failure in battery operation.

**Figure 5:**
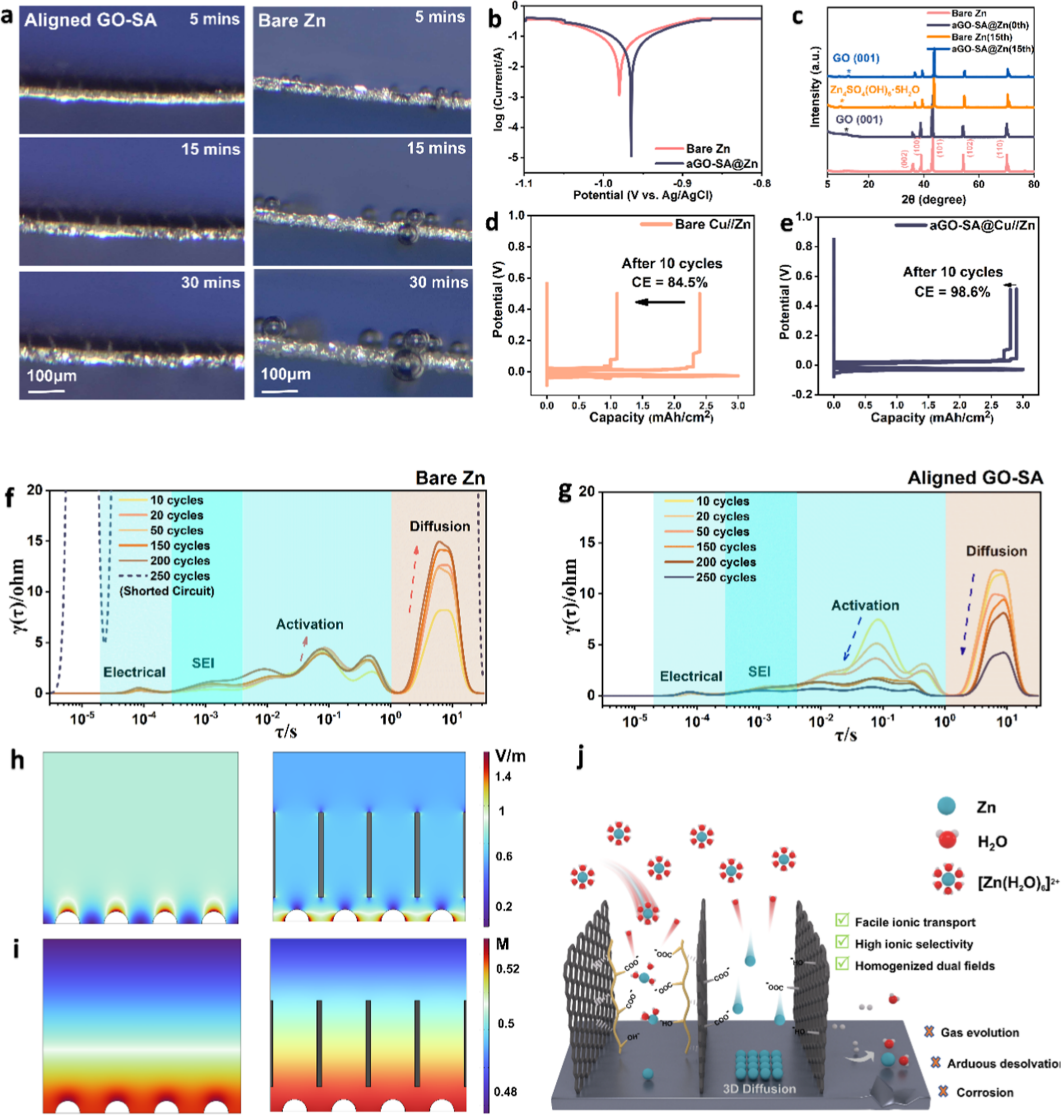
(a) In situ optical observation of (left) bare zinc under 5 mA·cm^–2^ current density for 30 min and (right) GO-SA-decorated zinc under 5 mA·cm^–2^ current density for 30 min. (b) Tafel plot measured from linear sweep voltammetry (LSV) indicating corrosion behaviors in 2 M ZnSO_4_; (c) ex-situ XRD patterns of pristine/coated samples before and after 15 cycles of cycling, with 1 mA·cm^–2^, 1 mA h·cm^–2^; the asterisk in orange implicates byproduct formation after cycling; the fabricated aGO-SA@Zn with numbers indicating different crystal planes of zinc and GO. (d) Evaluation of average CE of bare zinc and (e) aligned GO-SA@Cu on the Zn||Cu asymmetrical cell. DRT analysis of (f) bare zinc sample and (g) aGO-SA@Zn symmetrical cell after different cycles at 5 mA·cm^–2^, 2 mA h·cm^–2^. The dotted line means a short circuit of the cell. COMSOL transient simulation of current density distribution field and concentration field of (h) bare zinc and (i) aGO-SA@Zn, respectively. (j) Schematic diagram of biomimetic ion sieves as an effective way to protect zinc and prolong cycle life.

To evaluate the reversibility improvement by suppression of gas evolution, in situ optical cells are assembled and conducted. As shown in Figure 5a, successive deposition snapshots are captured with 10 mA·cm^–2^ current density for 30 min. The as-developed ion sieve in the left column shows a thickness of around 50 μm. The bush-like coating smoothly connects to the zinc surface and extends well into the electrolyte. During fast plating, zinc flux uniformly penetrates through the oriented channel and deposits at the composite electrode with a finely tuned desolvation and deposition texture. The coated sample is mostly intact, coupled with structural integrity and the absence of gas bubbles. In contrast, bare zinc is directly exposed to a mild acidic aqueous electrolyte and specifically adsorbs quantitative water molecules at the IHP. Under high cathodic potential, the fast plating with constrained Zn^2+^ desolvation kinetics would predictably cause a concentration gradient in both perpendicular and lateral directions, generating bubbles from competitive reductions of hydrogen ions. After 30 min of plating, the uneven deposition of zinc in gray color can be observed at both zinc edges and the main plate. The metallic dendrite leads to a vicious cycle due to the tip effect and would penetrate through the membrane to cause a short circuit. Overall, the in situ optical images firmly consolidate the suppressive effect of aligned coating on unrestricted dendrite growth and parasitic reactions.

Tafel extrapolation is performed to investigate the anticorrosion properties of the aligned GO-SA composite coating, where the anode is linearly polarized in the potential range of −1.1 to −0.8 V. From Figure 5b, it reveals that the coated sample shows a more positive corrosion potential of −0.965 V compared with −0.985 V of bare zinc. In terms of corrosion current, the coated sample shifts toward lower corrosion current (4.54 mA) as compared to the bare zinc sample (7.08 mA), indicating considerably improved corrosion resistance performance. LSV around hydrogen evolution potential is carried out to study the gas evolution behavior (Figure S17), and it shows that aligned GO-SA coating not only mitigates corrosion but also broadens the electrochemical stability window. At a current density of 10 mA·cm^–2^, the gas evolution potential of bare zinc (−1.76 V) shifts higher to −1.82 V, demonstrating an effectively extended cathodic potential window of hydrogen evolution, which is consistent with in situ microscope results and is expected to prolong cycle life. The ameliorated hydrogen evolution is largely owing to the selectivity of the aligned coating, which prohibits water molecules from adsorbing onto the electrode surface. As further proof, the radial distribution function (RDF) of water molecules at a compact layer (within 1 nm) is also calculated (Figure S18). Both bare zinc and coated zinc show peak density around 3 Å, characteristic of oriented water dipoles at the IHP. Notably, the coated sample has a comparatively lower distribution density, which consolidates the alleviated parasitic gas evolution from the aligned coating.^62^ XRD analysis is employed to spot the formation of byproducts during cycling. From Figure 5c, after 15 cycles, a distinct peak around 9° appears for the bare zinc sample, indicating the presence of Zn_4_SO_4_(OH)_6_·5H_2_O (ZHS). This insoluble byproduct not only consumes zinc ions that lead to capacity fading but also acts as a passivation layer on the anode surface. Being electrically inert, the layer impairs the interfacial reaction and significantly deteriorate the battery’s CE.^63^ In contrast, the aligned GO-SA-coated electrode shows minimal byproducts due to the zincophilic transport channels that provide sufficient active species and selective ion passage. As illustrated by MD simulation, water molecules and sulfate ions are restricted from approaching the surface, which also reduces byproduct formation.

The reversibility of zinc is further assessed by average CE based on a galvanostatic protocol on asymmetric Zn∥Cu cells.^64^ Specifically, the first formation cycle of the cell is conditioned by plating (1 mA·cm^–2^, 3 mA h·cm^–2^) and stripping Zn by charging to 0.5 V. Then, a Zn reservoir is deposited on a Cu electrode with an areal capacity of 3 mA h cm^–2^ (*Q*_t_) for subsequent cycling. Then, zinc stripping and plating at 1 mA h·cm^–2^ (*Q*_c_) are repeated for 9 cycles. In the last 10th cycle, the stripping capacity (*Q*_s_) is recorded at a final voltage of 0.5 V. Overall, the Zn plating/stripping CE is calculated by the equation shown in Figure S19. It can be calculated that the average CE of bare zinc||Cu is 84.5% after 10 cycles of stripping and plating, while for the coated Cu cell, that value can reach up to 98.6%. For Zn||Cu cells, further plating/stripping is carried out to evaluate the CE stability after long galvanostatic cycles at 1 mA·cm^–2^ and 0.5 mA h·cm^–2^, as shown in Figure S20. The copper foil with a composite coating presents a remarkably stable and high CE of over 99.5% after 2000 h. In contrast, the bare copper sample suffers from severe fluctuations of CE after 300 h of cycling, implicating drastic parasitic reactions and unstable charge transfer interfaces. This instability originates from dendrite growth, formation of dead zinc and gas evolution, which leads to low reversibility of bare zinc and ultimate severe battery failure.^65^ Voltage hysteresis reflects the degree of polarization as well as the energy barrier during cycling. According to Figure S21, the bare zinc sample shows obviously an enlarged gap between charging and discharging after 300 cycles, incrementing from 39.9 to 134.5 mV. This justifies increased internal resistance due to the accumulation of byproducts. For the prepared sample, the hysteresis value slightly increases, indicating stable reaction interfaces and a high utilization rate of active zinc ions, which contribute to reversible deposition and stripping.

The improved reversibility is further studied by the DRT tool, by which impedance is resolved during 250 cycles. As the cycle proceeds, bare zinc presents resistance increments at all time domains until dendrite penetration after 250 cycles, while the prepared sample witnesses gradually lowered impedance until the stabilized electrode–electrolyte interface (Figure 5g). Specifically, the formation of inert byproducts on bare zinc builds up an extra transfer barrier and manifests as remarkably increased diffusion (τ ≈ 7s) and charge transfer resistance (τ ≈ 0.5s and 0.01s).^66^ In quantity, diffusion impedance of bare zinc increases from 8.2 to 14.9 Ω, and electron transfer increments from 2.1 to 3.7 Ω after 200 cycles. It is also noteworthy that SEI impedance is also elevated by an insoluble passivation layer due to its electrical and ionic insulation at 0.001s. For prepared samples, the charge transfer resistance continuously decreases along cycling, coupled with a shift leftward to a shorter time constant, illustrating faster interfacial reaction kinetics and high CE.^62^ The cumulative and degenerative side reactions are largely influenced by the distribution of electric current and concentration of active species; thus, the advantage of aligned coating is revealed by dual-field transient simulation using COMSOL software. Regarding the current density field, Figure 5h illustrates that field strength intensifies at the nucleus seed on the bare zinc surface, where reactive species would be preferably attracted and gain electrons. The ionic distribution follows a similar pattern. The potential difference and concentration gradient stimulate rampant 2D solid-state diffusion, as previously discussed in chronoamperometry (CA). Lateral diffusion of Zn^2+^ species tends to take precedence over phase transformation, promoting dendrite growth. Furthermore, competitive gas evolution is also boosted by the tip effect, exacerbating battery degradation. For the aligned GO-SA sample, both current density and molar concentration distribution are regulated in a more uniform manner, benefiting from the finely grafted oriented structure. The high flux of Zn^2+^ and increased effective surface area within the channel ensure more uniform distribution and deposition, thereby improving zinc reversibility and significantly extending cycle life. The illustration diagram shown in Figure 5j summarizes the comprehensive benefits of a unidirectional ion sieve coating. The well-aligned skeleton creates a diffusion freeway with low tortuosity, enabling high ion flux and a minimized concentration gradient. Plus, the higher active area provides more available interactive sites, contributing to more readily nucleation and reaction kinetics. Dual-field homogeneity, as confirmed by finite element simulation, is also accomplished by structural modification. With natural polymer ingredients, the finely tuned surface chemistry of hybrid coating allows for stepwise desolvation and selective ion passage. Consequently, water-induced corrosion and parasitic reactions are effectively refrained, facilitating the cycling longevity of the vulnerable anodes in an aqueous environment.

### Electrochemical Performances

**Figure 6:**
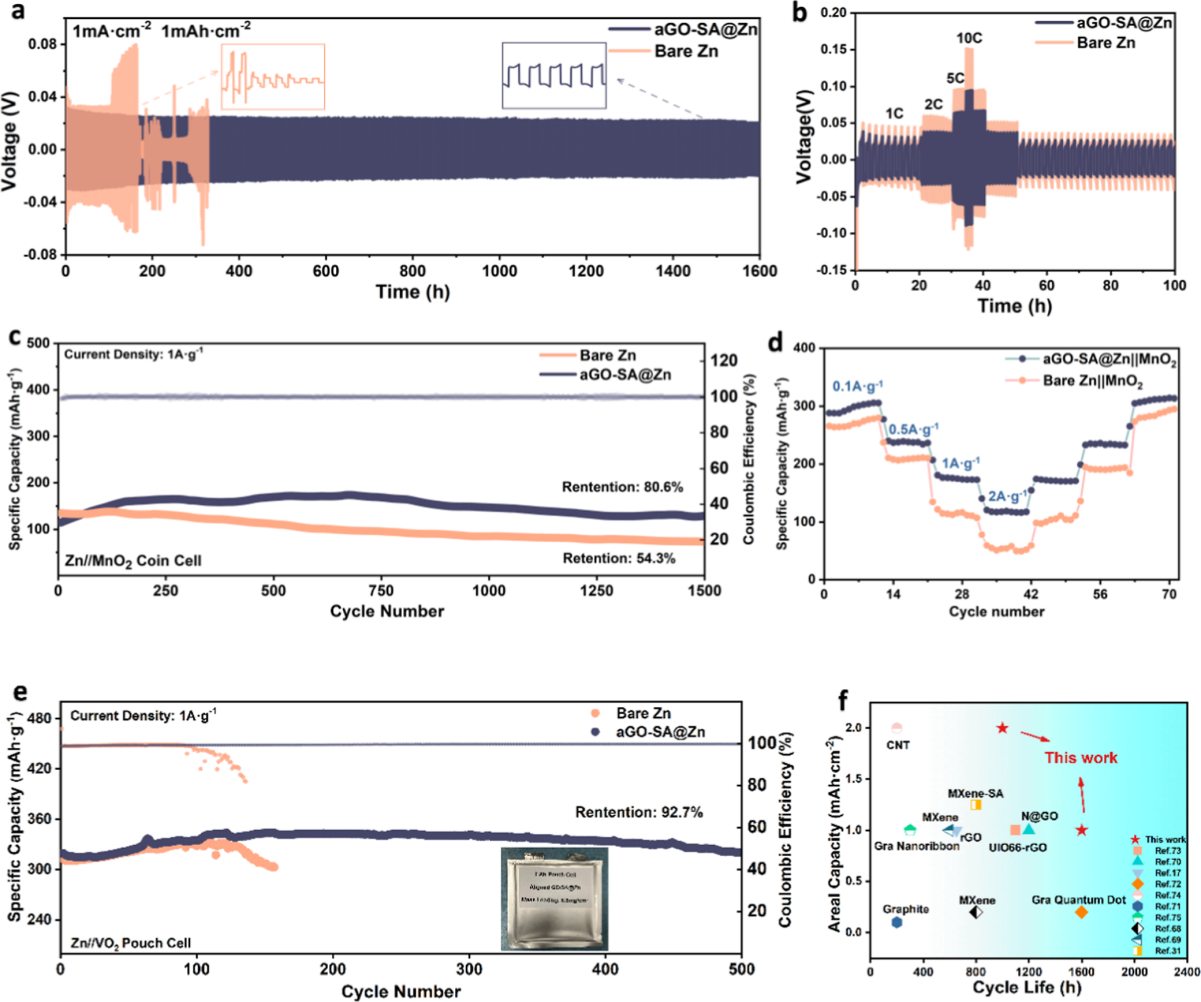
(a) Long-term galvanostatic cycling performance of bare zinc and aGO-SA@zinc symmetrical cells, with a current density of 1 mA·cm^–2^ and a capacity of 1 mA h·cm^–2^. (b) Rate tests of symmetrical cells at 1, 2, 5, and 10 mA·cm^–2^ with a fixed capacity of 1 mA h·cm^–2^. (c) Long-term cycling of a Zn||MnO_2_ coin cell with a current density of 1A·g^–1^. The colored lines refer to CE and specific capacity, respectively. (d) Rate performance of the Zn||MnO_2_ full cell with cumulative current densities at 0.1, 0.5, 1, and 2 A·g^–1^, respectively. (e) Cycling performance of a 7Ah Zn||VO_2_ pouch cell with an effective mass loading of 6.8 mg·cm^–2^. The inset presents the capture of the as-fabricated cell. (f) Comparison diagram of the cycle life with respect to different areal capacities in comparable literature.

The mitigated side reactions and improved reversibility of coated zinc are further consolidated by long-term galvanostatic cycling of the Zn||Zn symmetrical cell. At a current density of 1 mA·cm^–2^ and an areal capacity of 1 mA h·cm^–2^, the pristine zinc only sustains for 200 h before getting short-circuited, as shown in Figure 6a. The drastic instability implies erratic mass transfer interfaces with heterogeneous ion distribution and uncontrolled side reactions, which cause the consumption of solvent molecules and zinc dendrite penetration. By contrast, aligned GO-SA-coated zinc extends its life cycle to over 1600 h with negligible fluctuations, demonstrating uniform and stable zinc ion supplies. Moreover, voltage hysteresis is considerably reduced from 72 mV of bare zinc to 43 mV thanks to homogenized ion distribution, which also implies faster plating kinetics and more efficient energy utilization during cycling.^67^ At more hostile operating conditions of 5 mA·cm^–2^, 2 mA h·cm^–2^, the positive effect of aligned coating is distinctive with a life span of more than 1000 h, greatly exceeding that of bare zinc and comparable literature (Figure S22). The rate test provides direct evidence of enhanced reaction kinetics. As current density continuously increases to 10 mA·cm^–2^ during plating (Figure 6b), the energy-demanding desolvation and sluggish zinc transport start to lag behind, which causes greater concentration polarization overpotential in the short-term. As cycling proceeds, gas evolution and dendrite growth appear as major failure modes. The plating overpotential of the coated sample is 89 mV compared with 142 mV of bare zinc, which stands for a higher energy barrier and lower energy efficiency. This reduced plating barrier fully justifies the advantage of structural alignment in promoting ion transport kinetics. A cycling test with high depth of discharge (DoD) is also performed in Figure S23 to unveil the high stability and utilization efficiency of coated zinc (40 μm thickness). At 10 mA·cm^–2^ and 10 mA h·cm^–2^, namely, 42.7% DoD, reversible stripping, and plating are witnessed with extremely lower voltage hysteresis around 110 mV. As a parallel comparison, a performance chart is depicted. It presents life spans of the symmetrical cell in other literature with similar coating materials, including two-dimensional MXene sheets,^68,69^ nitrogen-doped graphene,^70^ graphite layers,^71^ graphene quantum dots,^72^ etc. Clearly, it can be observed that as-synthesized coated zinc also ensures competitive cycling life other than excellent transport kinetics.^73−75^

To demonstrate the improvement in practical usages, a Zn||MnO_2_ full cell is assembled as a coin cell with commercial electrolyzed MnO_2_. A CV test is also performed. As shown in Figure S24, both samples show characteristic redox peaks of Zn^2+^/H^+^ insertion and deinsertion (A1A2 and C1C2). Generally, it unveils that the aligned GO-SA group has obviously higher peak intensity and a larger area under the curve, referring to greater electrochemical activity due to increased zinc availability and an enlarged reactive surface. The potential gap between cathodic and anodic peaks of pure zinc is greater than the experimental group. Specifically, the oxidation peak of the aGO-SA group (A1) is 1.56 V, while it shifts higher to 1.63 V of the bare zinc group (A2). The CV result conforms to the symmetrical cell result where the aligned GO-SA group exhibits lower polarization and higher reversibility. The full cell rate test reveals similar patterns with the symmetrical cell, where the interfacial reaction of the decorated sample is less affected by increased concentration polarization. At a low current density of 0.1A·g^–1^, both samples deliver a high discharging capacity around 300 mA h·cm^–2^. However, the performance difference visibly broadens as current increases, which is caused by sluggish ion transport and elevated charge transfer resistance in bare zinc cells. For the long cycling test of the Zn||MnO_2_ cell at 1A·g^–1^ current density, both samples display an initial capacity around 125 mA h·cm^–2^. Nevertheless, the retention rate after 1500 cycles greatly differs. As ameliorated by homogenized concentration and electric fields, the occurrence of Zn^2+^ concentration gradient and dead zinc accumulation is effectively restrained for the aligned GO-SA sample. Therefore, a high capacity retention of 80.6% is achieved, compared with the bare zinc sample that merely retains 54.3% of the original capacity. Noteworthily, the high CE of coated zinc maintains above 99%, in witness of the high utilization rate and reversible stripping/plating of zinc species. This full cell cycling test again narrates the improved longevity and robustness of decorated zinc facilitated by the ion-sieving oriented channels.

As testimony for industrial practices, the as-synthesized aligned coating is coupled with the VO_2_ cathode to assemble a pouch cell, with a record high capacity of 7 Ah. The coated zinc electrode slice is shown in Figure S25, with an effective electrode area of 154.5 cm^2^ and an estimated N/P ratio of 4.7. For comparison, bare zinc with identical sizes is also assembled. It can be observed from the discharging curve that initial capacity reaches 320 mA h·cm^–2^, followed by activation of the cathode before reaching a peak capacity of 340 mA h·cm^–2^. At approximately 100 cycles, the bare zinc group displays obvious capacity degradation and sudden fluctuation of CE. Considering that cathode-induced capacity decay is primarily progressive,^76^ the zinc-derived failure mode thereby dominates. It is speculated that dendrites may cause a partial short circuit within the cell that leads to abrupt malfunction. Also, it could be the result of rampant gas evolution or byproduct passivation at the electrolyte–bare zinc interface, which accumulates to the extent where reactive species are blocked. For the coated sample, remarkable cycling stability for more than 500 h can be observed, with an impressive retention rate of 92.7%. The direct current internal resistance of discharging is evaluated to be 2 mΩ, which is comparable to that of commercial lithium-ion batteries. Overall, it can be concluded that the aligned GO-SA composite coating plays a vital role in promoting uniform ion transport and reaction kinetics as well as preventing uneven deposition and water-induced side reactions.

## Conclusions

In summary, we have developed a biomimetic aGO-SA composite layer anchored to Zn metal by a facile directional freezing method. Due to the distinctive characteristics and zincophilic properties achieved through “structural-functional” integration design, the unidirectional porous sieve offers remarkable advantages, including fast transport kinetics, extensive Zn^2+^ adsorption and selectivity, a homogenized dual-field distribution, and an improved EDL structure. MD simulation demonstrates enhanced Zn^2+^ transport kinetics due to reduced channel tortuosity, underpinned by an exceptionally high cation transference number of 0.85. The simulation also reveals anionic selectivity against SO_4_^2–^ to prevent potential byproduct formation. The incorporation of polar SA molecules maintains the porous and high-flux pathway while providing additional active sites for desolvation and deposition, minimizing the rate-limiting and energy-extensive electrochemical barriers. A low nucleation overpotential of 21 mV is thus achieved. DRT analysis investigates that while the coating does not markedly ameliorate electric charge transfer in the narrow context, it facilitates diffusion and selective absorption of Zn^2+^ instead. It also shows that solid-state migration during electron crystallization is effectively restrained, consistent with CA behavior, aiding in dendrite suppression. First-principles calculation reveals that Zn^2+^ species present greater affinity to the Zn(002) plane of coated Zn, compared with bare zinc. This explains the predominant planar deposition observed in the SEM and AFM morphologies. Ex situ and in situ characterizations on cycled samples illustrate fewer water-induced gas bubbles and byproducts on the aGO-SA@Zn sample, resulting in higher CE and extended cycle life. COMSOL dual-field simulation shows reduced electric tip effects and a more uniform ionic concentration field. Electrochemical tests validate the benefits of the aGO-SA coating, achieving dendrite-free and planar deposition for over 1600 h in the Zn half-cell, with minimal fluctuations with low voltage hysteresis below 40 mV. Even at a higher rate and capacity (5 mA·cm^–2^, 2 mA h·cm^–2^), its cycle life exceeds 1000 h. Additionally, a full pouch cell assembled with the VO_2_ cathode demonstrates a superior retention rate (over 90%) with a record high 7-Ah capacity. This work presents an effective strategy for anode modification in multivalent batteries and suggests its potential applicability as an anode design protocol with the simultaneously crafted structure and tuned interfacial chemistry.

## Methods

### Preparation of aGO-SA Composite Coating

GO is synthesized via a modified Hummer’s method. Then, GO solution (20 mL) and as-prepared SA glue (2 mL, 2.5 g·ml^–1^) are mixed with a volumetric ratio of 10:1 and undergo continuous stirring for 4 h to allow full mixing. Then, the mixture is evenly spread on a stainless-steel brick to form a 1 mm-thick supporter. Zn foil (40 μm) is tailored into samples with a diameter of 12 mm and placed on the surface of the coated supporter by mild pressure. In the next step, the stainless-steel supporter, together with the mixture, undergoes directional freezing in a homemade apparatus. The directional-frozen composite layer is then freeze-dried at 2 Pa for 48 h using a YTLG-10 freeze drier. After that, the composite layer can be readily peeled off from the stainless steel brick by spontaneously sticking to zinc foil. The as-obtained zinc foil with a GO-SA (denoted aGO-SA) membrane can be directly used to assemble coin cells.

### Preparation of the Cathode

For the fabrication of Zn||MnO_2_ full batteries, electrolyzed manganese dioxide is used and mixed with SuperP Li and poly(vinylidene fluoride) (PVDF) in a ratio of 7:2:1 with NMP as the solvent. The slurry goes through doctor-blade coating onto 316L stainless steel with a diameter of 12 mm at a mass loading of around 1.2 mg cm^–2^. Anodes and cathodes were assembled in a 2032 cell with glass fiber as the separator and 180 μL of a 2 M ZnSO_4_ electrolyte.

## Supporting Information Available

Material synthesis details; cell fabrication details; characterization equipment including SEM, XRD, FTIR, and XPS; electrochemical testing protocol such as CV, impedance, Tafel, DRT, etc., computational and simulation details, including DFT, MD, and COMSOL; a schematic diagram of the fabrication of a coating; SEM of graphene joints; GO-SA interaction model; a side view image of the aGO-SA coating; soaking experiment; activation energy calculation; adsorption model for DFT calculation; transference number calculation; O 1s XPS spectra; Zn 2p XPS spectra; CV curve at different scan rates; Cottrell correlation; DRT results at different deposition capacities; top SEM image after cycling; ex-situ XRD results; adsorption energy calculation model; HER scanning test; RDF from MD calculation; CE calculation; galvanostatic cycling curve of the Zn||Cu asymmetrical cell; voltage hysteresis curve; galvanostatic cycling at 5, 2 mA·cm^–2^; high DoD test; CV curve of full cell; pouch cell; COMSOL modeling grid; and COMSOL simulation for random coating (PDF)
MD animation for aligned coating (MP4)
MD animation for random coating (MP4)
