# Supplementary material for: Unidirectional Ion Sieve Enabling High-Flux and Reversible Zinc Anodes
Source: ACS Nano. 2025 Apr 8;19(15):14987–5001. doi: 10.1021/acsnano.5c01103 (PMC12020423; doi:10.1021/acsnano.5c01103)
Supplement: Supplementary file 1 — nn5c01103_si_001.pdf [file nn5c01103_si_001.pdf]

Supplementary Information

for

Unidirectional ion sieve enabling high-flux and reversible zinc anodes

Zhiyuan Chen,[1] Yifan Zhao,[1,2] Ping Cui,[1] Jiayan Zhu,[3] Xuan Gao,*[4,5] Guanjie He,*[4] Xiaosu Yi*[1]

[1] Faculty of Science and Engineering, The University of Nottingham Ningbo China, Ningbo 315100, China

[2] Department of Energy Storage Center, Shanghai Advanced Research Institute, Chinese Academy of Sciences, 99 Haike Road, Shanghai, China

[3]State Key Laboratory of Superhard Materials, College of Physics, Jilin University, Jilin, Changchun, 130012, PR China

[4] Christopher Ingold Laboratory, Department of Chemistry, University College London, 20 Gordon Street, London, WC1H 0AJ, UK

[5] Thom Building, Department of Engineering Science, University of Oxford, 17 Parks Road, Oxford, OX1 3PJ, UK

Experimental Method

Graphene oxide is synthesized via modified Hummer's method, which is previously reported elsewhere. Briefly, 325mesh graphite flakes (3g) are added in to a mixture of concentrated H_2SO_4 (98wt%, 69ml) and $NaNO_3$(1.5g) while maintaining the mixture temperature below 10℃. Then $KMnO_4$ (9g) as oxidizing agent is carefully added into the previous hybrid solution step by step. For a couple of minutes of violent oxidation, water (138ml) is then introduced to produce exotherm to 98℃, followed by termination of reaction with 3ml H_2O_2 (30wt%) solution. Then the oxidized graphene oxide layers are washed and centrifuged to obtain thick GO solution. Graphite powder is purchased from Qingdao Xintanyuan Co.Ltd., while the involved strong acid and $NaNO_3$ of AR grade are purchased from Sigma Aldrich. Sodium alginate AR is purchased from Xilong Scientific. GO precursor (6mg/ml) is prepared by diluting the as-synthesized thick solution. Sodium alginate with 1wt% concentration is

stirred overnight to form homogeneous glue. Then GO solution (20ml) and as-prepared SA glue (2ml) is mixed with volumetric ratio of 10:1 and undergoes continuous stirring for 4 hours to allow full mixing. Then the mixture is evenly spread on a stainless-steel brick to form a 1 mm-thick supporter. Zn foil (40μm), which are tailored into samples with diameter of 12 mm and placed on the surface of the coated supporter by mild pressure. In the next step, the stainless-steel supporter together with mixture are subjected to directional freezing from its bottom surface in contact with a copper disk to its top surface in a homemade apparatus. The directional-frozen composite layer is then freeze-dried at 2Pa for 48 hours using YTLG-10 freeze drier. After that, the composite layer can be readily peeled off from stainless steel brick by spontaneously sticking to zinc foil. The as-obtained zinc foil with GO-SA (denoted aGO-SA) membrane can be directly used to assembly coin cells. For random aligned GO-SA coated zinc, all procedures are identical except that the sample endures vacuum drying instead of directional freeze drying. For symmetric batteries, Zn, GO-SA@Zn and aGO-SA@Zn plates served as electrodes on both sides. These samples are assembled in 2032 coin cell with 2 M ZnSO4 electrolyte addition of 180 μL; Two slices of GFA glass fiber (Whattman, 290um thick) are used as separators. For fabrication of Zn||MnO_2 full batteries, electrolyzed manganese dioxide is used and mixed with SuperP Li and Polyvinylidene fluoride (PVDF) in a ratio of 7:2:1 with NMP as solvent. The slurry goes through doctor-blade coating onto 316L stainless steel with diameter of 12 mm at a mass loading of around 1.2 mg cm^{-2}. Anodes and cathodes were assembled in a 2032 cell with glass fiber as the separator and 180 μL of 2 M ZnSO4 electrolyte. Pouch cells are assembled with respect to both bare zinc sample and aligned GO-SA coated sample. The double-side coated cathode material is VO_2(B) with effective mass loading of 6.8mg·cm^{-2}, which is provided by Zhejiang Vastech Energy Co., Ltd. The size of an anode is approximately 13×12cm, with an overhang of 2mm smaller than cathode. The battery case used is commercially available aluminum plastic film. A total of 10 layers of cathode and 11 layers anode is compacted with aluminum clamp for perpendicular confinement.

The morphologies and microstructures of the aGO-SA hybrid membrane is observed with a ZEISS scanning electron microscope (SEM) with EHT of 10kV. GO and MnO_2 are characterized using a Rigaku D/Max 2500 X-ray diffractometer (XRD), Thermo Fisher Escalab 250 X-ray photoelectron spectroscopy (XPS). FITR is tested using Nexus 670 model. Contact angle was obtained on OCA 15Pro. Electrochemical tests are conducted using a symmetric cell configuration by assembling the aGO-SA zinc anode or Zn electrodes into 2016-type coin cells at room temperature. The electrolytes used are 2 M ZnSO4 aqueous solution, and the electrolyte amount applied in the cells was ~30μL. GFD glass fiber is used as separators. To explore the electrochemical behavior of Zn foils, galvanostatic charging-discharging cycling is performed on Neware testing platform at a current density of 1 mA cm^{-2} and a total capacity of 1 mAh·cm^{-2}. For electrochemical tests such as cyclic voltammetry (CV) and Impedance test (EIS) is tested with model Chi660E workstation with operating range of 0.8-1.9V. Tafel extrapolation is performed with conventional three-electrode configuration with the as-prepared anode as working electrode, platinum plate

as the counter electrode and Ag/AgCl as the reference electrode in electrolyte of 2.0 M ZnSO4.

DFT computations are performed using VASP code[1], which applies the Perdew–Burke–Ernzerhof (PBE) functional within the generalized gradient approximation (GGA).[2] The projector-augmented wave (PAW) pseudopotential[3] with a kinetic energy cutoff of 500eV describe the expansion of electronic eigenfunctions. Vacuum thickness of 25 Å is chosen to minimize inter-layer interactions. Brillouin-zone integration utilized a Γ-centered 5 × 5 × 1 Monkhorst–Pack k-point sampling. Atomic positions were fully relaxed before energy and force converged to tolerances of 1×10^{-5}eV and 0.03 eV/Å, respectively. To account for long-range interactions, the dispersion-corrected DFT-D method is employed.[4] The adsorption energy (E_{ads}) between two molecules, A and B, forming a complex, can be determined by the equation: $E_{ads} = E_{complex} - (E_a + E_b)$, Where: $E_{complex}$ represents the total energy of the A-B molecular complex. E_A and E_B denote the total energies of isolated molecules A and B, respectively. The MD simulations are performed in the NVT ensemble using the LAMMPS code with a 1fs timestep.[5] Trajectory visualization and analysis were carried out using the Ovito software. The system consists of graphene oxide, sodium alginate, water, sulfate ion and zinc ion. The OPLS-AA force field is applied for graphene oxide and sodium alginate.[6, 7] The water model uses Tip4p/spc. Hydrogen atomic positions are kept rigid with the SHAKE and RATTLE algorithms.[8] The non-bonded van der Waals interactions are modeled using the 12-6 Lennard-Jones potential, while electrostatic interactions such as long-range Coulomb interactions, are addressed by the particle-particle-particle mesh technique.[9] Both the random and aligned GO-SA simulation boxes have size of 8.0 nm × 7.5 nm × 36.1nm and 2.2 nm; while the experimental sample contains nine graphene oxide sheets and two sodium alginate molecules, subsequently giving a total of 17247 atoms. In the production run, 0.1 fs is set as the time step, and the data are collected every 1 ps. The system is minimized (atomic positions and cell sizes), keeping the box length isotropic. For each system, two independent trajectories of 1000 ps are generated. All production runs are under microcanonical ensemble at 298 K and 1 bar pressure for 10 ps to equilibration. Then a microcanonical ensemble at 298 K is performed to obtain the parametric of the system for 2000 ps. For dual field simulation, COMSOL Multiphysics 6.1 is used. The grid for bare zinc, random coating and vertical coating are presented in S13. Briefly, the grid size is 8μm × 8μm in total; The total current density of the model was set to 3.0 mA cm-2, the diffusion coefficient for both Zn^{2+} and SO_4^{2-} are set as 2×10^{-9} m/s. Initial ion concentration is selected as 500 mol/m^3. The exchange current density is 22.5mA • cm^{-2}. All presented results are captured after 1s of simulation.

Distribution of relaxation times (DRT) serves as analytical tools for studying ion dynamics from EIS measurements. The detailed DRT data were computed using MATLAB R2022b with the 'DRT Tools' package, developed by Francesco Ciucci's group.[10] The full-width at half-maximum (FWHM) coefficient was set to 0.3 for shape control.

For electrochemical measurements, galvanostatic charge-discharge (GCD) is tested with NEWARE testing systems. Linear polarization (LSV) and Tafel measurements are conducted with a three-electrode system, where bare zinc or coated zinc serves as the working electrode.

The counter electrode and reference electrode used are Ti plate and Ag/AgCl, respectively. Both CV and EIS are tested with Chi660E electrochemical workstation, where EIS testing frequency ranges from 0.1Hz to 10^5Hz; The nucleation overpotential is measured with asymmetrical coin cell. For the blank group, it uses Zn//bare Cu coin cell, while for the experimental group it measures on Zn//aGO-SA coated Cu. The aGO-SA coated Cu is fabricated following identical procedure with aforementioned aGO-SA@Zn sample. The discharging (deposition) capacity is set as 3 mAh·cm^{-2}with current density of 1 mA·cm^{-2}. The nucleation overpotential is measured at first cycle. For Coulombic efficiency tested with Zn//Cu asymmetrical cell, aGO-SA is coated on copper foil with identical fabrication process to that of aGO-SA@Zn. EDL capacitance can be obtained from C=i_{EDL}/v, where C is the capacitance, i_{EDL} is the double layer current, v is the scan rate. Here, we select i_{EDL} as an absolute average of current difference during the forward scan and negative scan at 0 V.

Reaction mechanism between GO-SA and zinc, as proposed by other literature:[11]

$$Zn + GO + 2H^{+} = Zn^{2+} + rGO + H_2O$$

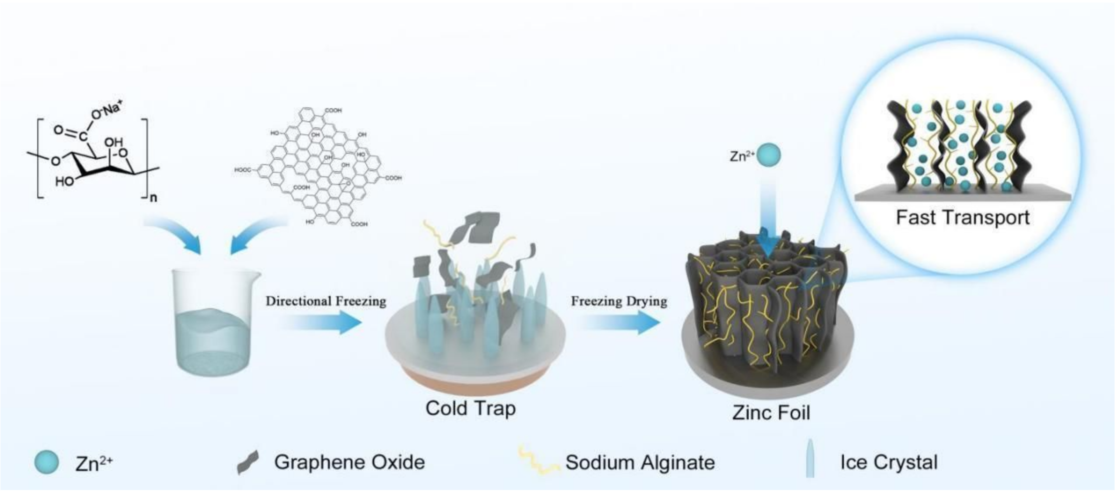

Figure S1. *Schematic Diagram of the fabrication process of aligned GO-SA composite film with ice-assisted template method. The oriented microstructure of aGO-SA coating is inserted in the magnified image from cross-sectional view.*

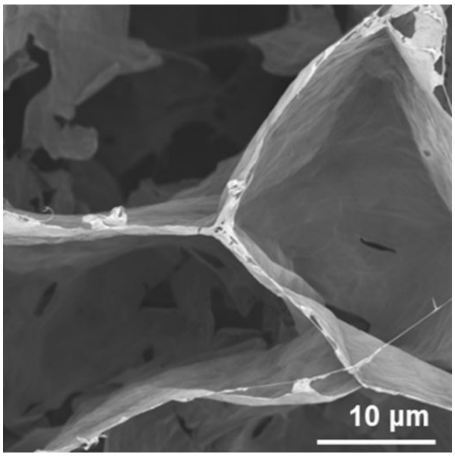

Figure S2. *Top view magnified images of the joint parts between sheets.*

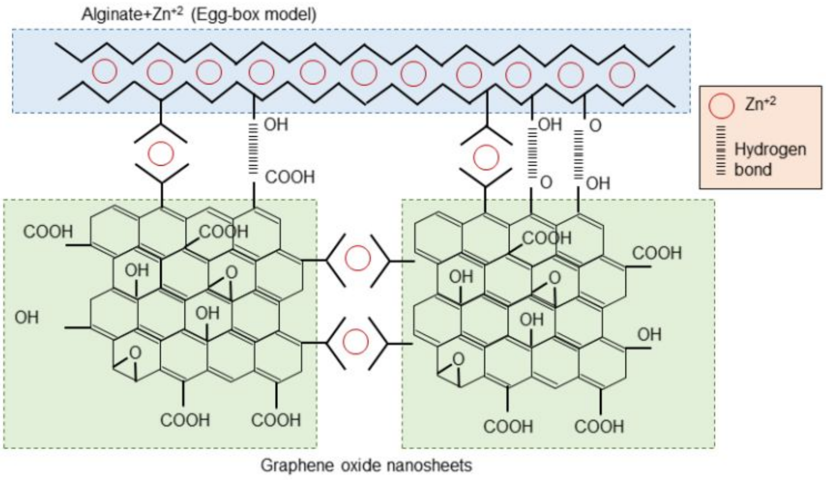

Figure S3. *The egg-box model to explain interaction between alginate and* Zn^{2+}*,as well as the interactions between graphene oxide sheets and sodium alginate through hydrogen bonds.*[12]

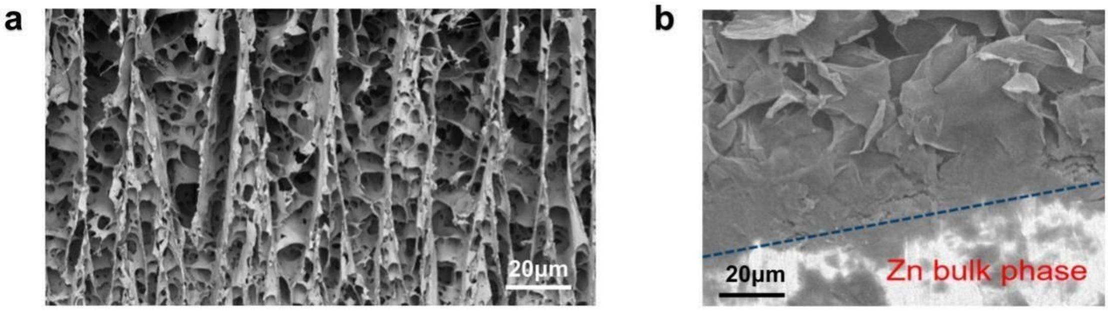

Figure S4. *a) Side-view SEM images of structural continuity of the coating, which maintains uniform for more than 100um. b) Side-review SEM image at the coating-zinc boundary.*

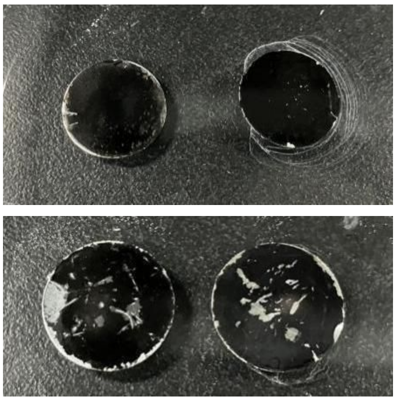

Figure S5. *Photos of top) aGO-SA composite coating; bottom) pure GO coating after soaking in 2M $ZnSO_4$ for 48 hours.*

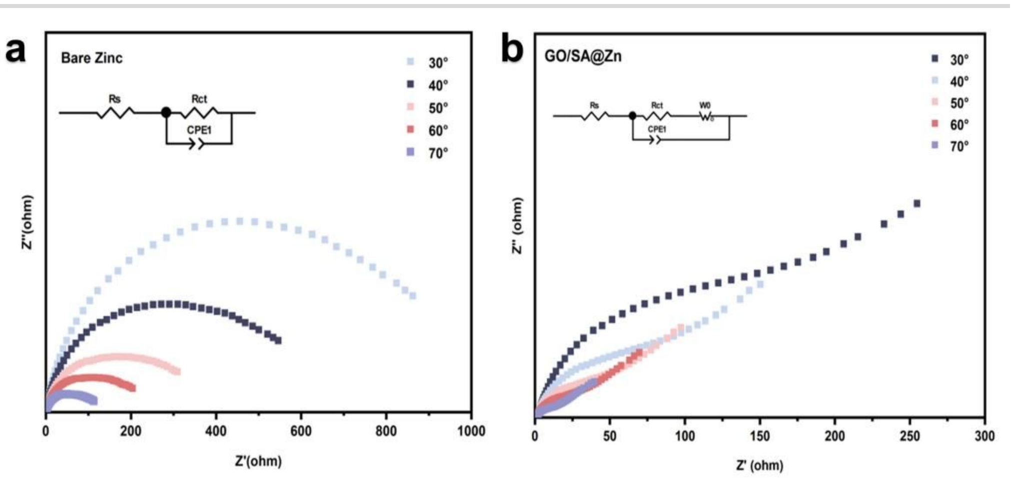

Figure S6. *EIS diagram of a) Bare zinc and b) aGO-SA@Zn at different temperatures for activation energy (E_a) calculation.*

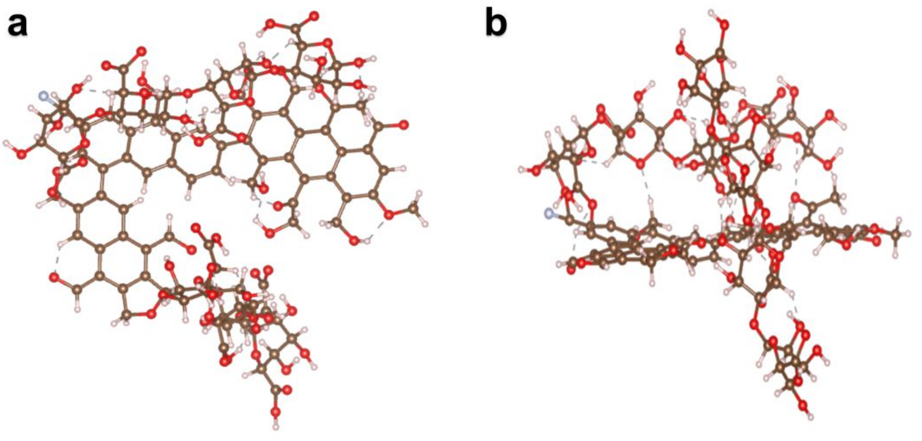

Figure S7. *aGO-SA models in adsorption energy calculation using DFT. a) Top view and b) Side view.*

The transfer number of Zn^{2+} (t_{Zn2+}) is calculated by the following equation:

$$t_+ = \frac{I_{ss}(\Delta V - I_0 R_0)}{I_0(\Delta V - I_{ss} R_{ss})}$$

where ΔV is the pulse voltage applied, I_0 and R_0 are respectively the initial current and resistance, while Is and Rs are the steady-state current and resistance. The calculated results and iA diagram are shown as follows.

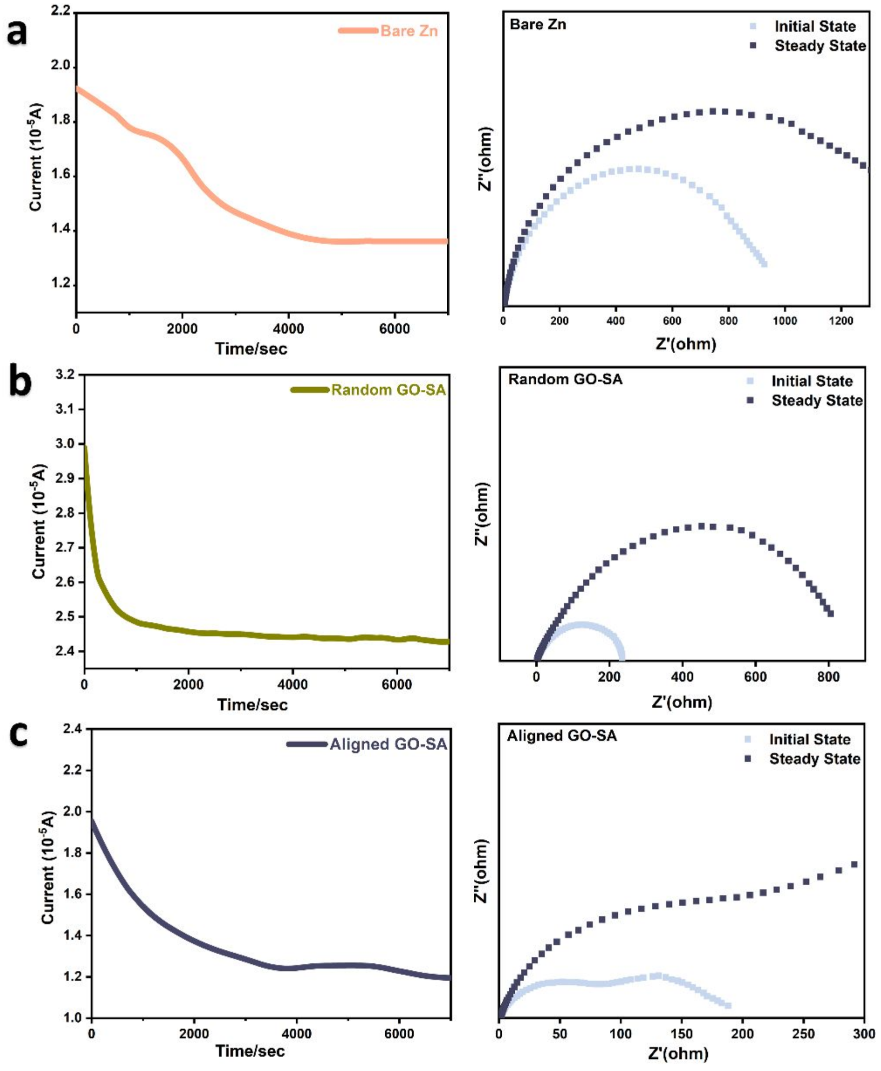

	ΔV	I_0 (mA)	I_{ss} (mA)	R_0 (Ω)	R_{ss} (Ω)
Bare Zn	0.005	0.019	0.014	669.9	961.3
Random	0.005	0.03	0.024	188.9	636.7
Aligned	0.005	0.022	0.012	73.8	240

Figure S8. *Calculation equation of cation transfer number. iA curve and EIS at initial and steady states for* ***a)*** *bare zinc and* ***b)*** *Random GO-SA@Zn and* ***c)*** *Aligned GO-SA@Zn. The chart at the bottom tabulates corresponding parameters for transference number calculation.*

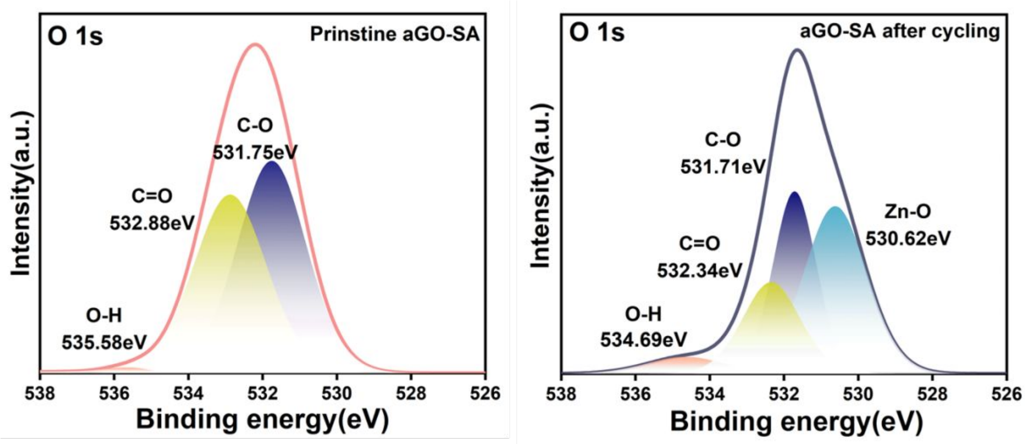

Figure S9*. O1s XPS spectra of bare zinc and aGO-SA@Zn after 150 cycles at 1mA • cm^{-2},1mAh • cm^{-2} ; The colored region indicates differentiation of O at various oxidation states, coupled with respective bond type.*

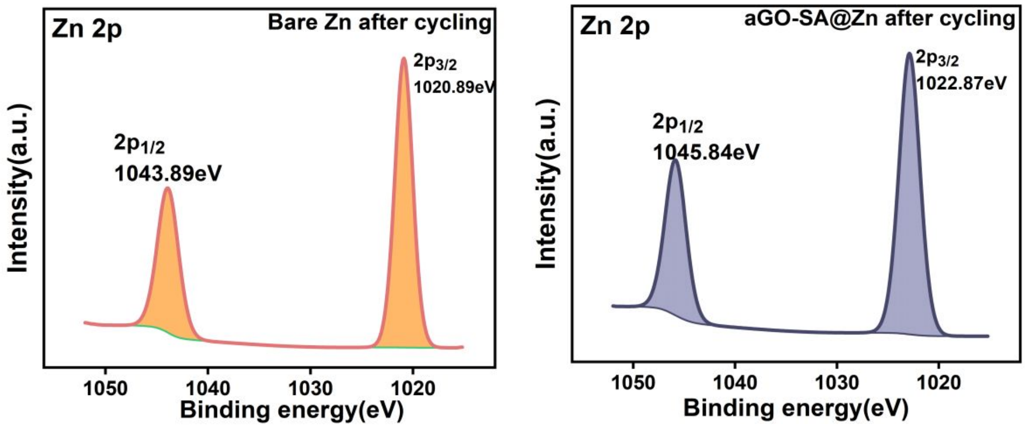

Figure S10*.Zn 2p XPS spectra of bare zinc and aGO-SA@Zn after 150 cycles at 1mA • cm^{-2} 1mAh • cm^{-2}.*

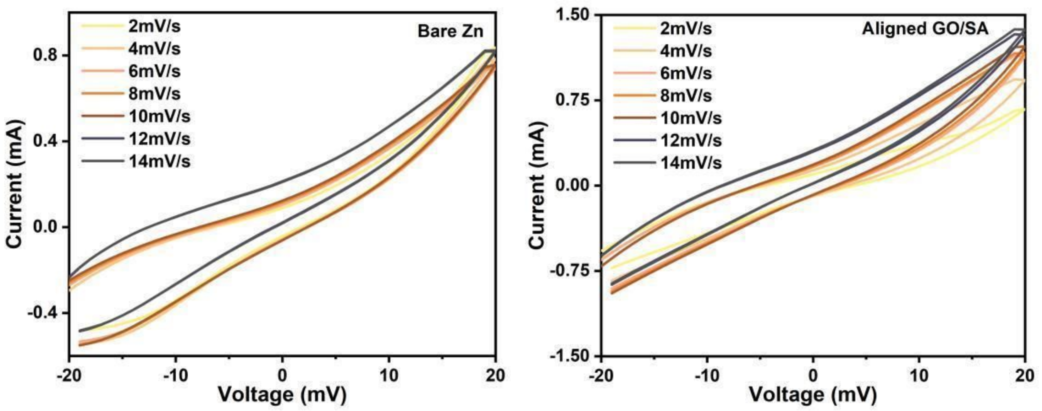

Figure S11. *CV curves of left) Bare zinc and right) GO-SA coated zinc at different scanning rates*

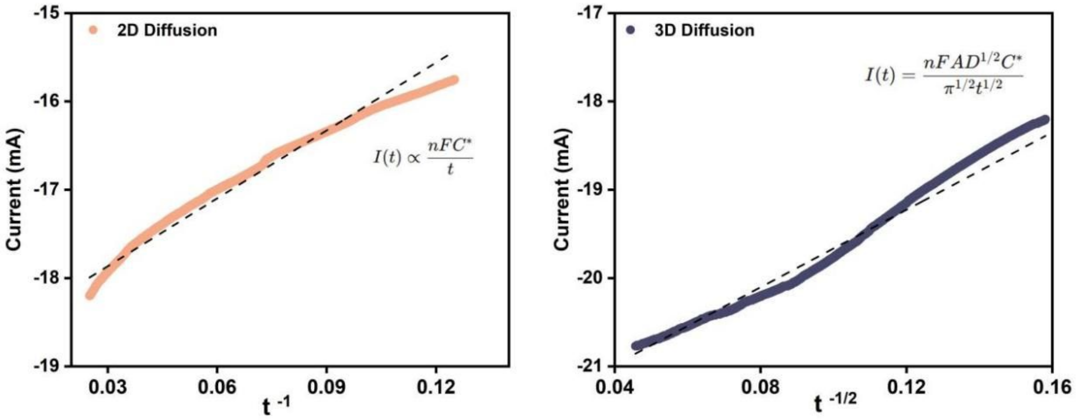

Figure S12. *Correlation of chronoamperometry curve of aligned GO-SA coated zinc by Cottrell equation for* ***left)****2D diffusion and* ***right)****3D diffusion.*

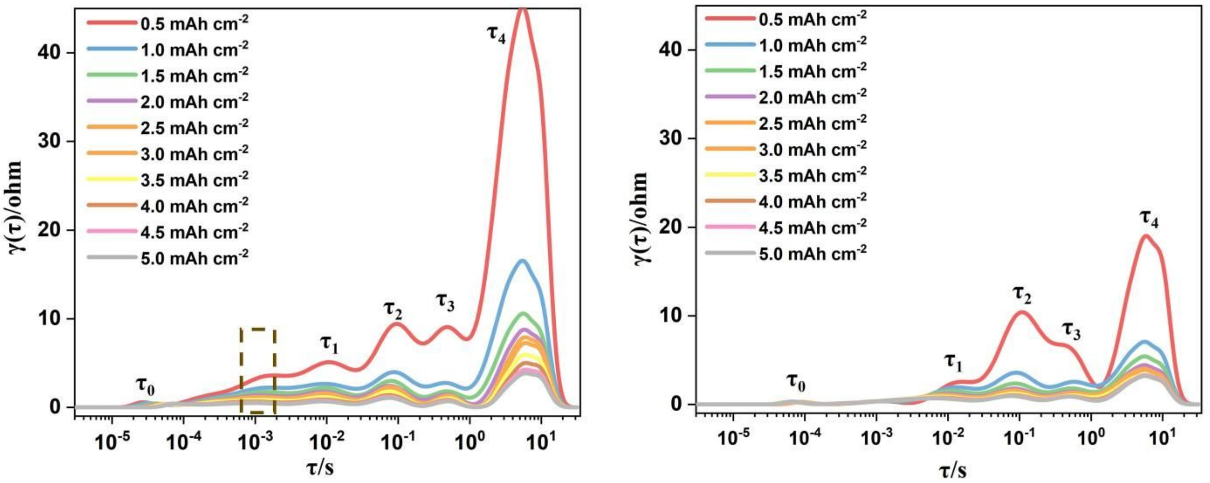

Figure S13. *DRT results of* ***left)****bare zinc sample and* ***right)****aligned GO-SA sample with a total capacity of 5mAh • cm^{-2} at current density of 5mA • cm^{-2}. Tests are performed at each consecutive 0.5mAh • cm^{-2}. Annotations refer to characteristic time constant representing different electrochemical processes.*

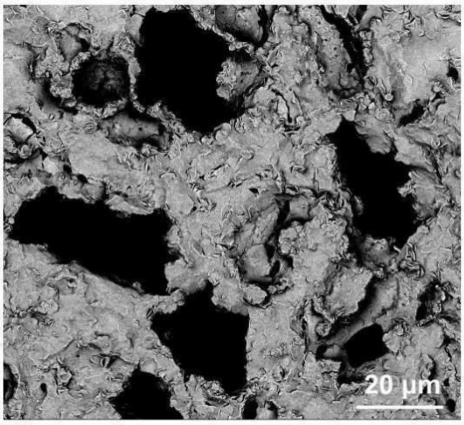

Figure S14. *Top SEM images of the coated sample after cycling, where unidirectional pores retain.*

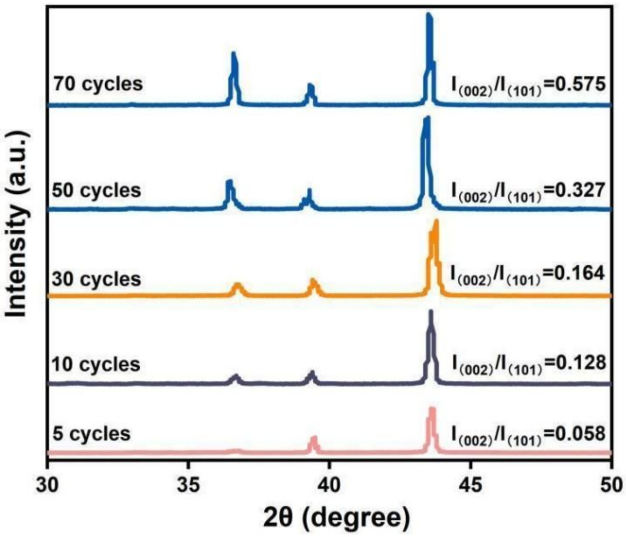

Figure S15. *Ex-situ XRD results of aligned GO-SA sample after removal of the coating, with different cycles at 1mA • cm^{-2} 1mAh • cm^{-2}.*

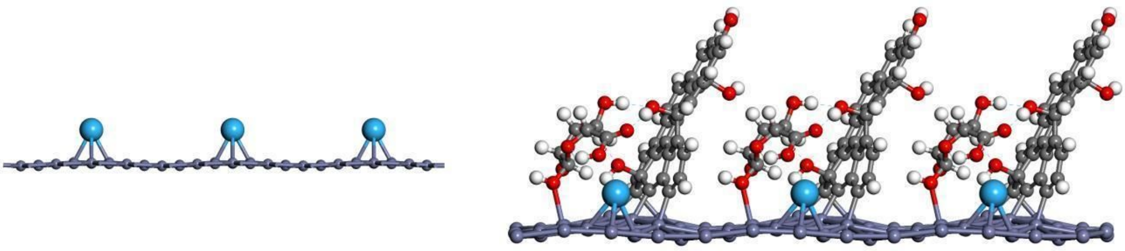

Figure S16. *DFT calculation models: adsorption energy of Zn^{2+} towards bare zinc (left) and Zn (002)-GO/SA composite (right).*

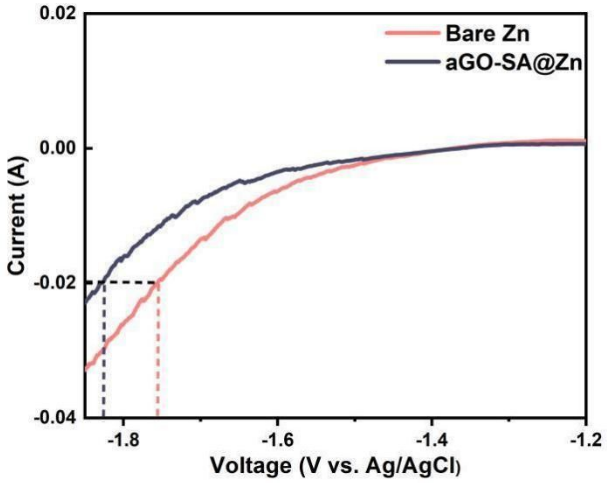

Figure S17. *Hydrogen evolution (HER) scanning tested from Zn//Ti symmetrical cells, where 1M $NaSO_4$ electrolyte with scan rate of 5mV. Characteristic current value is chosen as 20mA.*

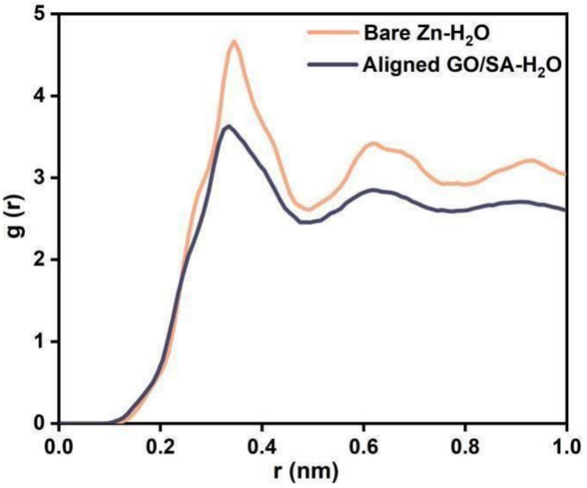

Figure S18. *Radial Distribution Function (RDF) of bare Zn-H_2O and coated Zn-H_2O using MD simulation.*

$$CE = \frac{(9Q_c + Q_s)}{(9Q_c + Q_t)}$$

The standard protocol 'reservoir half-cell' for assessing average Coulombic Efficiency (CE) by Zn//Cu asymmetrical cell. Specifically, the steps start from first conditioning cycle with 3mAh • cm^{-2} capacity deposition and then stripping, aiming to diminish substrate influence. Then a zinc reservoir is built on the Cu electrode with fixed capacity of 3mAh • cm^{-2}(Q_t). After that, a current density of 1mA • cm^{-2} is applied for subsequent stripping and plating with 1mAh • cm^{-2}(Q_c). After 9 cycles of repetition, the final stripping capacity (Q_s) is recorded after plating with cut-off voltage 0.5V.

Figure S19. *Coulombic Efficiency calculation from standard protocol.*[13]

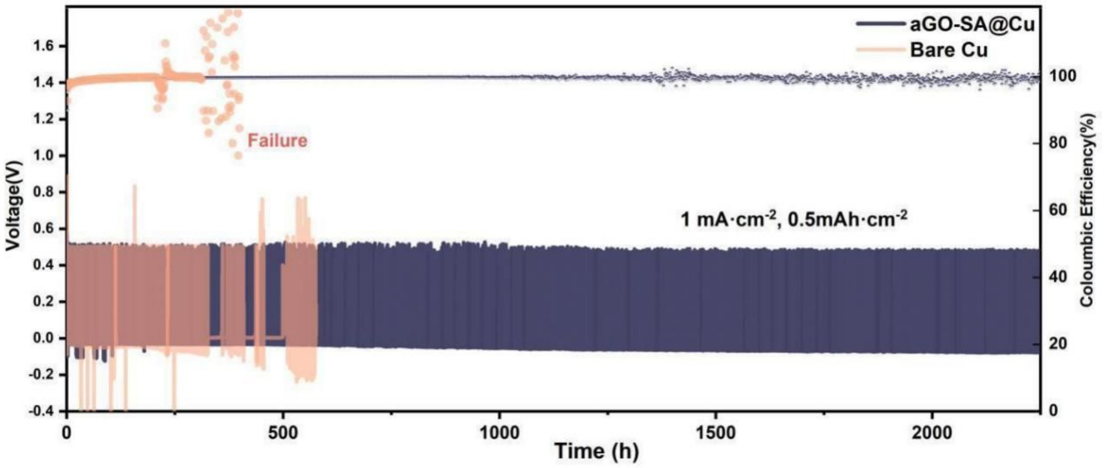

Figure S20. Bottom) *Galvanostatic charging - discharging profiles of Zn||Cu asymmetric cells at 1mA • cm^{-2} 0.5mAh • cm^{-2};* ***Top)*** *Coulombic efficiency of bare Cu and aGO-SA@Cu respectively.*

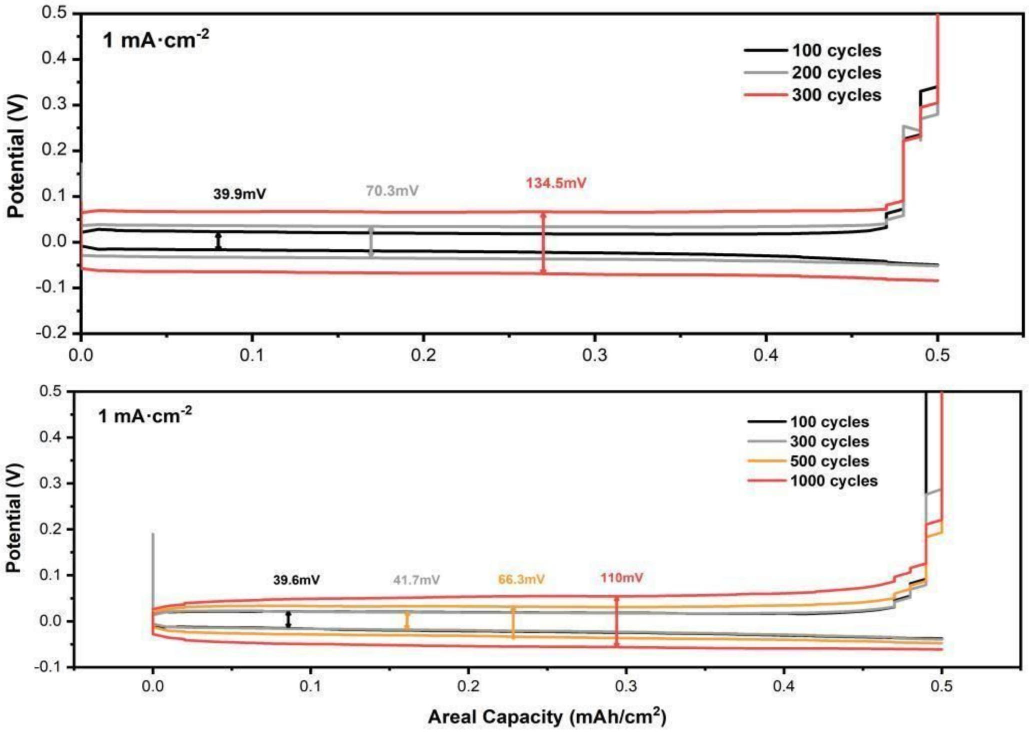

Figure S21. *Voltage hysteresis evolution of Zn//Cu asymmetrical cell for different cycles at 1mA • cm^{-2}; Top: Bare zinc after 100,200,300 cycles; Bottom: aligned GO-SA@Zn after 100,300,500,1000 cycles.*

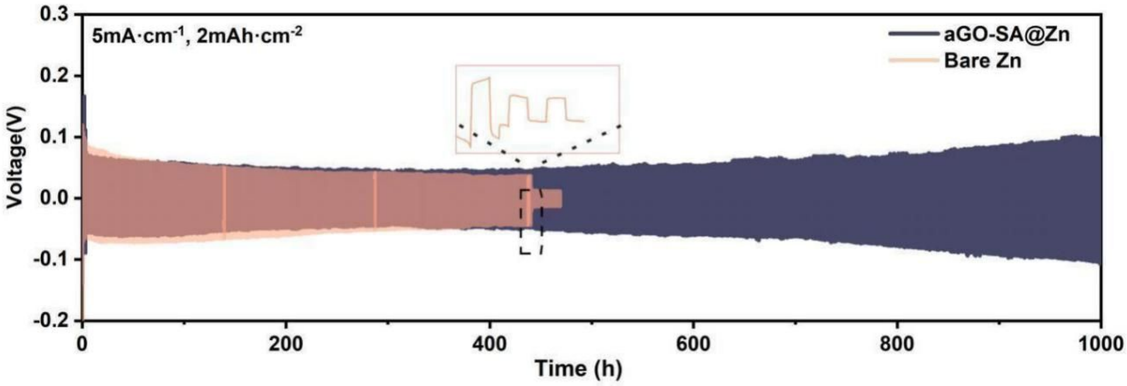

Figure S22. *Galvanostatic cycling at 5mA • cm^{-2} 2mA • cm^{-2} for both samples.*

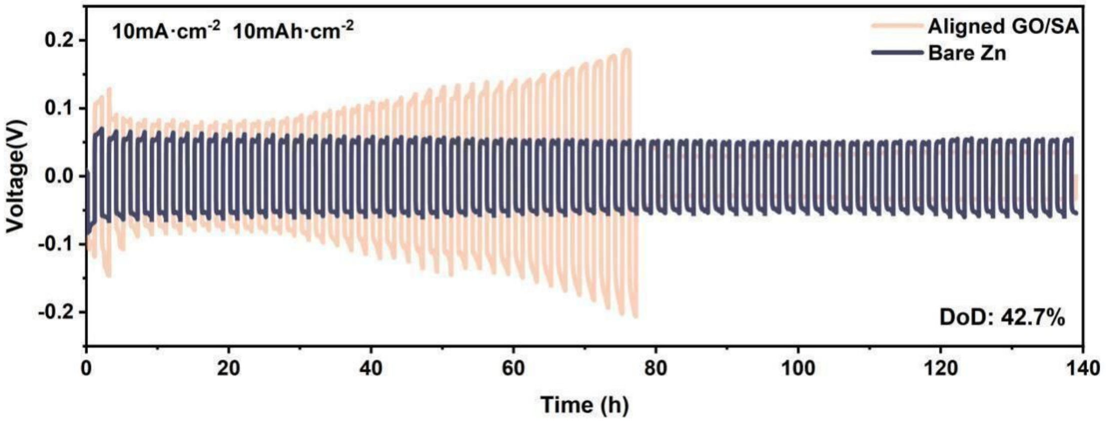

Figure S23. *High depth discharge at 10mA • cm^{-2} 10mA • cm^{-2} for both samples.*

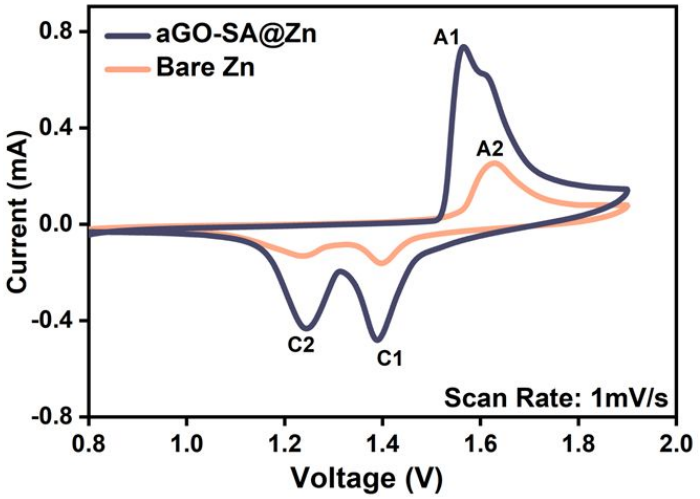

Figure S24. *Cyclic voltammetry (CV) curve of bare and decorated Zn||MnO_2 full cell with scan rate of 1mV/s.*

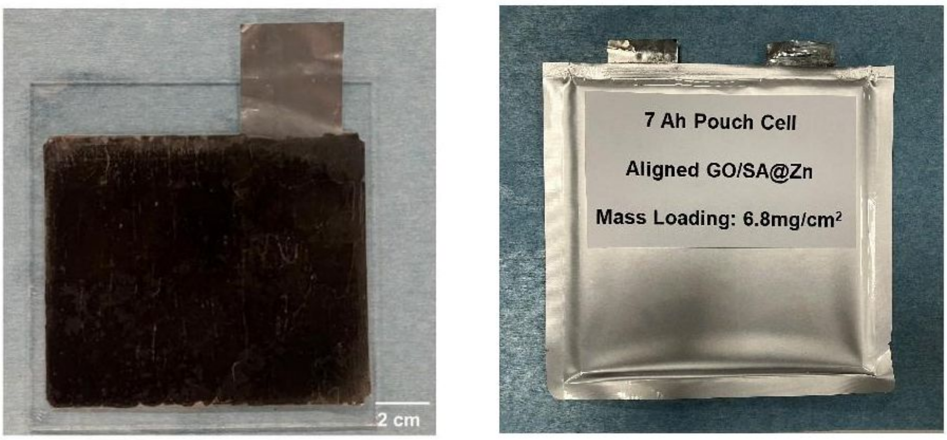

Figure S25. *Image of coated zinc anode of pouch cell, with anode size around 13 ×12cm. Also, the 7Ah pouch*

cell is shown on the right-hand side.

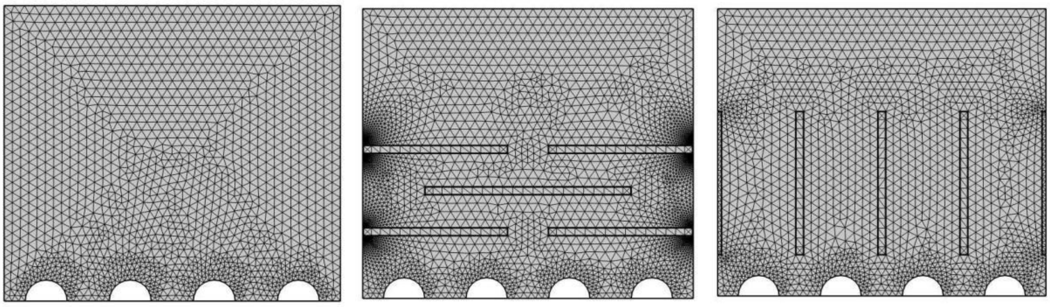

Figure S26. *Simulation grid in COMSOL Multiphysics.* ***left)*** *Grid for bare zinc electrode; middle) for random GO-SA coating and* ***right)*** *grid for aGO-SA coating.*

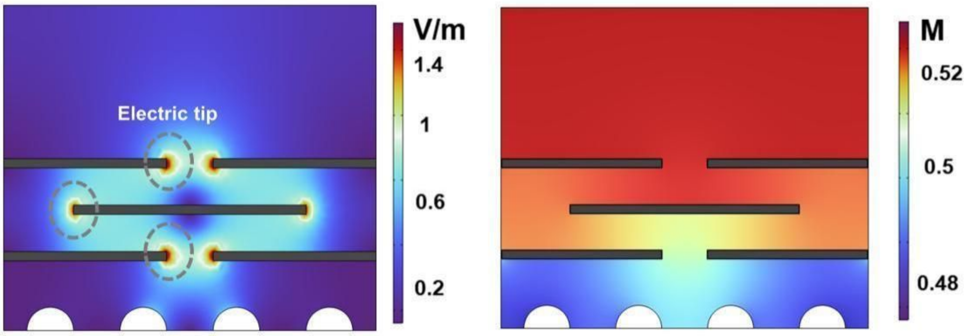

Figure S27. *COMSOL simulation result of random GO-SA coating, in terms of electric field (left) and concentration field (right).*

References

(1) Kresse, G.; Furthmüller, J. Efficiency of ab-initio total energy calculations for metals and semiconductors using a plane-wave basis set. *COMP MATER SCI* **1996**, *6* (1), 15-50.

(2) Perdew, J. P.; Burke, K.; Ernzerhof, M. Generalized gradient approximation made simple. *PRL* **1996**, *77* (18), 3865.

(3) Blochl, P. E. Projector augmented-wave method. *Phys. Rev., B Condens* **1994**, *50* (24), 17953-17979.

(4) Grimme, S. Semiempirical GGA-type density functional constructed with a long-range dispersion correction. *J. Comput. Chem.* **2006**, *27* (15), 1787-1799.

(5) Plimpton, S. Fast parallel algorithms for short-range molecular dynamics. *J. Comput. Phys.* **1995**, *117* (1), 1-19.

(6) Shaari, N.; Kamarudin, S.; Basri, S. Molecular dynamics simulations of sodium alginate/sulfonated graphene oxide membranes properties. *Heliyon* **2018**, *4* (9).

(7) Sun, H. Ab initio calculations and force field development for computer simulation of polysilanes. *Macromolecules* **1995**, *28* (3), 701-712.

(8) Andersen, H. C. Rattle: A "velocity" version of the shake algorithm for molecular dynamics calculations. *J. Comput. Phys.* **1983**, *52* (1), 24-34.

(9) Hockney, R. W.; Eastwood, J. W. *Computer simulation using particles*; crc Press, 2021.

(10) Wan, T. H.; Saccoccio, M.; Chen, C.; Ciucci, F. Influence of the discretization methods on the distribution of relaxation times deconvolution: implementing radial basis functions with DRTtools. *ELECTROCHIM ACTA* **2015**, *184*, 483-499.

(11) Xia, A.; Pu, X.; Tao, Y.; Liu, H.; Wang, Y. Graphene oxide spontaneous reduction and self-assembly on the zinc metal surface enabling a dendrite-free anode for long-life zinc rechargeable aqueous batteries. *Appl. Surf. Sci* **2019**, *481*, 852-859.

(12) Sabater i Serra, R.; Molina-Mateo, J.; Torregrosa-Cabanilles, C.; Andrio-Balado, A.; Meseguer Dueñas, J. M.; Serrano-Aroca, Á. Bio-Nanocomposite hydrogel based on zinc alginate/graphene oxide: Morphology, structural conformation, thermal behavior/degradation, and dielectric properties. *Polymers* **2020**, *12* (3), 702.

(13) Ma, L.; Schroeder, M. A.; Borodin, O.; Pollard, T. P.; Ding, M. S.; Wang, C.; Xu, K. Realizing high zinc reversibility in rechargeable batteries. *Nat. Energy* **2020**, *5* (10), 743-749.
